# Female Gene Networks Are Expressed in Myofibroblast-Like Smooth Muscle Cells in Vulnerable Atherosclerotic Plaques

**DOI:** 10.1161/ATVBAHA.123.319325

**Published:** 2023-08-17

**Authors:** Ernest Diez Benavente, Santosh Karnewar, Michele Buono, Eloi Mili, Robin J.G. Hartman, Daniek Kapteijn, Lotte Slenders, Mark Daniels, Redouane Aherrahrou, Tobias Reinberger, Barend M. Mol, Gert J. de Borst, Dominique P.V. de Kleijn, Koen H.M. Prange, Marie A.C. Depuydt, Menno P.J. de Winther, Johan Kuiper, Johan L.M. Björkegren, Jeanette Erdmann, Mete Civelek, Michal Mokry, Gary K. Owens, Gerard Pasterkamp, Hester M. den Ruijter

**Affiliations:** 1 Laboratory of Experimental Cardiology (E.D.B., M.B., E.M., R.J.G.H., D.K., M.D., H.M.d.R.), University Medical Centre Utrecht, Utrecht University, the Netherlands.; 2 Central Diagnostic Laboratory (L.S., M.M., G.P.), University Medical Centre Utrecht, Utrecht University, the Netherlands.; 3 Department of Vascular Surgery (B.M.M., G.J.d.B., D.P.V.d.K.), University Medical Centre Utrecht, Utrecht University, the Netherlands.; 4 Robert M. Berne Cardiovascular Research Center (S.K., G.K.O.), University of Virginia, Charlottesville.; 5 Center for Public Health Genomics (R.A., M.C.), University of Virginia, Charlottesville.; 6 Department of Biomedical Engineering (M.C.); 7 University of Virginia, Charlottesville (M.C.).; 8 Institute for Cardiogenetics, University of Lübeck, Germany (R.A., T.R., J.E.).; 9 A.I. Virtanen Institute for Molecular Sciences, University of Eastern Finland (R.A.).; 10 Experimental Vascular Biology, Department of Medical Biochemistry, Amsterdam University Medical Centers — location AMC, University of Amsterdam, Netherlands (K.H.M.P., M.P.J.d.W.).; 11 Division of BioTherapeutics, Leiden Academic Centre for Drug Research, Leiden University, the Netherlands (M.A.C.D., J.K.).; 12 Department of Genetics and Genomic Sciences, Icahn School of Medicine at Mount Sinai, New York (J.L.M.B.).; 13 Department of Medicine, Karolinska Institutet, Karolinska Universitetssjukhuset, Huddinge, Sweden (J.L.M.B.).

**Keywords:** coronary artery disease, gene expression, lipids, plaque, women's health

## Abstract

**BACKGROUND:**

Women presenting with coronary artery disease more often present with fibrous atherosclerotic plaques, which are currently understudied. Phenotypically modulated smooth muscle cells (SMCs) contribute to atherosclerosis in women. How these phenotypically modulated SMCs shape female versus male plaques is unknown.

**METHODS:**

Gene regulatory networks were created using RNAseq gene expression data from human carotid atherosclerotic plaques. The networks were prioritized based on sex bias, relevance for smooth muscle biology, and coronary artery disease genetic enrichment. Network expression was linked to histologically determined plaque phenotypes. In addition, their expression in plaque cell types was studied at single-cell resolution using single-cell RNAseq. Finally, their relevance for disease progression was studied in female and male *Apoe^−/−^* mice fed a Western diet for 18 and 30 weeks.

**RESULTS:**

Here, we identify multiple sex-stratified gene regulatory networks from human carotid atherosclerotic plaques. Prioritization of the female networks identified 2 main SMC gene regulatory networks in late-stage atherosclerosis. Single-cell RNA sequencing mapped these female networks to 2 SMC phenotypes: a phenotypically modulated myofibroblast-like SMC network and a contractile SMC network. The myofibroblast-like network was mostly expressed in plaques that were vulnerable in women. Finally, the mice ortholog of key driver gene *MFGE8* (milk fat globule EGF and factor V/VIII domain containing) showed retained expression in advanced plaques from female mice but was downregulated in male mice during atherosclerosis progression.

**CONCLUSIONS:**

Female atherosclerosis is characterized by gene regulatory networks that are active in fibrous vulnerable plaques rich in myofibroblast-like SMCs.

**Highlights**

- Gene regulatory networks in advanced carotid female atherosclerotic plaques point to dominant smooth muscle cell biology.
- Myofibroblasts seem to play a major role in shaping female atherosclerosis.
- Expression of a myofibroblast-associated gene regulatory network in women is associated with an unfavorable plaque phenotype.

Women are predominantly protected from cardiovascular disease at young ages. However, once in their 70s, the incidence of coronary artery disease (CAD) in women surpasses that of men.^1^ This suggests a strong interaction of sex and age,^2,3^ where menopause is a known inflection point.^4^

Pathology studies have described 2 major mechanisms in acute coronary syndromes: plaque rupture and superficial plaque erosion. The latter are most prevalent in women^5^ but also contribute substantially to acute coronary syndromes in younger men.^6,7^ In the past decades, most attention has been focused on understanding plaque rupture hallmarked by large atheroma and plaque hemorrhage. Plaques with superficial erosion on the other hand are often characterized by their fibrous composition and lack of substantial calcification.^8^ Despite the clinical importance of symptomatic fibrous plaques in the era of lipid-lowering therapy,^9^ the mechanisms that lead to fibrous plaques and erosion have received less attention.^10^

The complexity of atherosclerosis presents several challenges that can be addressed by molecular phenotyping of the affected vascular tissue.^11–13^ In complex systems such as atherosclerotic plaques, genes are organized in functional networks (Table 1). These networks contain highly connected key driver (KD) genes, which are responsible for maintaining network connectivity and function.^14^ The connectivity of these networks (ie, how strongly connected the genes in the network are) can be seen as a proxy for how active a network is (network activity).^15,16^ This systems biology approach has provided great insight into our understanding of disease mechanisms active in CAD.^11^

**Table 1.**
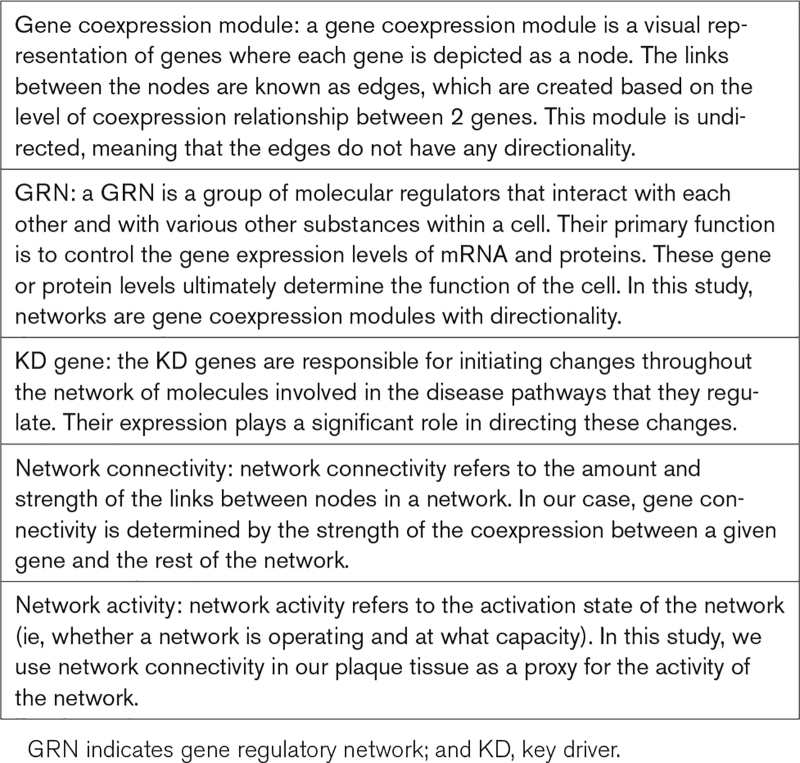
Common Concepts in Systems Biology Studies

We applied this systems biology approach in atherosclerotic tissues of female patients with CAD and identified 3 female-biased gene regulatory networks (GRNs, blue, yellow, and greenyellow). These networks were female biased, had higher expression in diseased tissue compared with healthy mammary artery as well as evidence of genetic relevance for CAD.^17^ The expression of these female GRNs was the highest in smooth muscle cells (SMCs; blue), endothelial cells (ECs; yellow), and macrophages (greenyellow).^17^ Endothelial to mesenchymal transition (EndMT) is considered a potential mechanism of plaque erosion^18^ and was found enriched in the blue female-specific GRN.^17^ Furthermore, genes identified as KDs from the network primarily expressed in SMCs. These KD genes were upregulated in female atherosclerotic tissues compared with male atherosclerotic tissue. In *Klf4* (KLF transcription factor 4) knockout lineage-tracing mice,^19^ these KD genes were expressed in phenotypically modulated SMCs, suggesting that these genes play a role in SMCs’ plasticity in female plaques.^17^

Despite these recent advances, it is unknown how these female GRNs contribute to plaque phenotypic differences between sexes. We hypothesize that female GRNs are associated with plaque characteristics and predict plaque vulnerability in women. Therefore, we applied an unbiased systems biology approach to male and female atherosclerotic plaques from the carotid artery. We prioritized 2 female GRNs that are active in ECM (extracellular matrix)-producing myofibroblasts and involved in vulnerable plaque phenotypes. In line with these findings, we observed that KD gene *MFGE8* of the female myofibroblast network remained expressed in plaques isolated from lineage-tracing female mice during atherosclerosis progression but were downregulated in plaques from male mice.

## MATERIALS AND METHODS

### Data and Scripts Availability

Anonymized data and materials have been made publicly available at the Dataverse NL and can be accessed at https://doi.org/10.34894/4IKE3T, https://doi.org/10.34894/TYHGEF, and https://doi.org/10.34894/D1MDKL. The scripts that support the findings of this study are available from the corresponding author upon reasonable request.

### Human Patient Samples

Patients undergoing endarterectomy of the carotid artery in 2 Dutch tertiary referral centers between 2002 and 2020 were included in this study. Study procedures comprise of a baseline blood withdrawal, an extensive questionnaire filled in by the participants verified against medical records, and collection of carotid arterial plaque material during surgery. All patients provided written informed consent before surgery; the study was approved by the Local Medical Ethical Committee and conducted according to the Declaration of Helsinki.^20^ Details of the Athero-Express study protocol have been described previously.^21–23^ All available plaque bulk RNA female samples were used in this study; male samples were downsampled by splitting the male samples in the 10-year groups and selecting random men in each group to match the number of women in the same age groups.

### Mice

The University of Virginia Animal Care and Use Committee approved animal protocols (protocol 2400). The Cdh5-Cre ERT2 R26R-eYFP Apoe*^−/−^* mice (species: *Mus musculus*, age: 6 weeks at the time of tamoxifen treatment, sex: 13 men and 9 women, strain: C57BL/6J, source: The Jackson Laboratory) used in the present study have been described in our recent study.^24^ The animals were randomized after genotyping at the age of 6 weeks before starting the experiment. All experiments were performed blindly. Cdh5 Cre ERT2 R26R-eYFP Apoe*^−/−^* mice, Cre recombinase was activated with a series of 10 tamoxifen injections (1 mg/d per mouse; Sigma-Aldrich, T-5648) over a 2-week period. One week after the tamoxifen treatment, mice were switched from a normal chow diet (Harlan Teklad TD.7012) to a high-fat Western diet (WD), containing 21% milk fat and 0.15% cholesterol (Harlan Teklad; TD.88137) for 18 or 30 weeks.

### Atherosclerotic Plaque Morphometry of Mouse Brachiocephalic Artery Lesions

Paraformaldehyde-fixed paraffin-embedded brachiocephalic arteries (BCAs) were serially cut into 10-μm-thick sections from the aortic arch to the bifurcation of the right subclavian artery. For morphometric analysis, we performed modified Russell-Movat staining on the BCA at 750 µm. The lesion, lumen, external elastic lamina (outward remodeling), and internal elastic lamina area were measured on digitized images of the Movat staining using Fiji software, version 1.53c. For lesion area, lumen area, external elastic lamina area, and internal elastic lamina area, 1-way ANOVA method in GraphPad Prism, 9.4.1 version was used.

### Statistical Analysis

Statistics were performed using the R statistical software. Data normality was determined using the Kolmogorov-Smirnov test. For comparison of 2 groups of continuous variables with normal distribution and equal variances, 2-tailed unpaired Student *t* tests were performed with a confidence level of 95%. For comparison of 2 groups of continuous variables with normal distribution and unequal variances, 2-tailed unpaired Student *t* tests followed by a Welch correction were performed with a confidence level of 95%. Two-tailed unpaired Mann-Whitney *U* test with a confidence level of 95% was conducted if data were non-normally distributed.

### Human Plaque Histology

As described previously,^3,21,23^ the atherosclerotic plaque was processed directly after surgery, and (immune-)histochemical staining was routinely performed on the culprit lesion (segment with the highest plaque burden) for identification of macrophages (CD68), calcification (hematoxylin-eosin), SMCs (alpha actin), collagen (picro sirius red), plaque hemorrhage (hematoxylin-eosin, Elastin von Gieson staining), vessel density (CD34), and fat (picro sirius red, hematoxylin-eosin). Assessment of overall plaque vulnerability was performed as previously described by Verhoeven et al.^21^ Briefly, macrophages and SMCs were semiquantitatively defined as no/minor or moderate/heavy. Each plaque characteristic that defines a stable plaque (ie, no/minor macrophages, moderate/heavy collagen, moderate/heavy SMCs, and <10% fat) was given a score of 0, while each plaque characteristic that defines a vulnerable plaque (ie, moderate/heavy macrophages, no/minor collagen, no/minor SMCs, and ≥10% fat) was given a score of 1. The score of each plaque characteristic was summed resulting in a final plaque score ranging from 0 (most stable plaque) to 4 (most vulnerable plaque). Intraobserver and interobserver variability were examined previously and showed good concordance (κ=0.6–0.9).^25^

### Bulk RNA Sequencing of Human Carotid Plaques

As the culprit lesion is used for plaque histology following the standardized Athero-Express protocol,^21^ the adjacent plaque segments were used for RNA sequencing. To measure bulk RNA expression in the plaques, total RNA was isolated according to the manufacturers protocol after processing of the plaque segments using ceramic beads and tissue homogenizer (Precellys, Bertin Instruments, Montigny-le-Bretonneux) with use of TriPure (Sigma-Aldrich). After precipitating RNA in the aqueous phase with propanolol, RNA was washed with 75% ethanol and either used immediately after an additional washing step with 75% ethanol or stored in 75% ethanol for later use. Subsequently, library preparation was performed as described before.^26–28^ Ethanol was removed and the pellet air-dried. Then, primer mix (5 ng primer per reaction) was added to initiate primer annealing at 65 °C for 5 minutes. Subsequently, reverse transcription was executed. Subsequent reverse transcription reaction; first strand reaction for 1 hour at 42 °C, heat inactivated for 10 minutes at 70 °C, second strand reaction for 2 hours at 16 °C, and then put on ice until proceeding to sample pooling.

This initial reverse transcription reaction used the following primer design: an anchored polyT, a unique 6-bp barcode, a unique molecular identifier of 6 bp, the 5′ Illumina adapter, and a T7 promoter, as described (57). Each sample now contained its own unique barcode making it possible to pool together cDNA (complementary DNA) samples at 7 samples per pool. cDNA was cleaned using AMPure XP beads (Beckman Coulter), washed with 80% ethanol, and resuspended in water before proceeding to the in vitro transcription reaction (AM1334; Thermo-Fisher) incubated at 37 °C for 13 hours. Next, Exo-SAP (Affymetrix, Thermo-Fisher) was used to remove primers, upon which amplified RNA was fragmented, cleaned with RNAClean XP (Beckman Coulter), washed with 70% ethanol, air-dried, and resuspended in water. RNA yield and quality in the suspension were checked by Bioanalyzer (Agilent) after removal of the beads with use of a magnetic stand. By performing a reverse transcription reaction using SuperScript II reverse transcriptase (Invitrogen/Thermo-Fisher) according to the protocol of the manufacturer, cDNA library construction was initiated. Next, polymerase chain reaction amplification was performed as described previously.^26–28^ Polymerase chain reaction products were cleaned twice using AMPure XP beads (Beckman Coulter). Qubit fluorometric quantification (Thermo-Fisher) and Bioanalyzer (Agilent) were used to check Library cDNA yield and quality. Illumina Nextseq500 platform was used to sequence the libraries; paired end, 2×75 bp. After sequencing, retrieved fastq files were de-barcoded and split into forward and reverse reads. From there, the reads were mapped using Burrows-Wheel aligner (BWA37), version 0.7.17-r1188, calling bwa aln with settings -B 6 -q 0 -n 0.00 -k 2 -l 200 -t 6 for R1 and -B 0 -q 0 -n 0.04 -k 2 -l 200 -t 6 for R2, bwa sampe with settings -n 100 -N 100, and a cDNA reference (assembly hg19, Ensembl release 84). Read and unique molecular identifier counts were acquired from SAM files with use of custom perl code and collected into count matrices. Further analyses were performed using R,^29^ version 3.6.2 and later and its IDE Rstudio,^30^ version 1.2 and later. Genes were annotated with Ensembl ID’s, basic quality control was performed (filtering out samples with low gene numbers [<10 000 genes] and read counts [<18 000 reads]).

### GRN Analysis in Human

The software package WGCNA^31^ (v. 1.69) was used to generate modules of coexpression genes on the 486 available men RNAseq samples. After excluding all the ribosomal genes and including only the protein-coding genes with annotated HGCN names, a set of 12 765 protein-coding genes, which passed quality control (average >1 count per sample) was used for module generation. The raw read counts were corrected for unique molecular identifier sampling:


$$\begin{array}{l} Corrected\;count=-4096*\ln\left(1-\frac{Raw\;count}{4096}\right) \end{array}$$

, then normalized by sample sequencing depth and log-transformed. A signed network was constructed using the robust bicor correlation measure. To determine the exponent used for the adjacency matrix construction, soft thresholding analysis was performed with the WGCNA package for powers ranging from 2 to 30. The cutoff for assuming scale-free topology was set at an R^2^ of 0.8, while having a median connectivity of lower than 100. The reason for using these cutoffs was 2-fold: first, to conform with the scale-free topology requirement for the creation of the networks, this topology has been shown to be a useful representation of biological pathways.^32,33^ Second, to conform with the scale-free topology criteria previously established in Hartman et al^17^ for comparability between the networks. The chosen lowest complying power was 24 (Figure S1). We performed a sensitivity analysis and show that other soft-threshold powers only marginally changed the grouping of the studied modules (Table S1). The network was constructed by first generating an adjacency matrix, which was transformed into a topological overlay matrix.^31^ Modules were detected by clustering the average distance of the dissimilarity matrix (defined as 1-topological overlay matrix) and cutting the subsequent dendrogram by using the *cutreeDynamic* function (deepSplit=3, minClusterSize=20). Modules were named after colors as provided by the WGCNA package, for each analysis, Gray module was excluded as it is considered a bin module where genes that cannot be assigned to a module are placed. Module eigengenes were calculated by taking the first principal component of gene expression in that module. The module eigengenes were correlated to clinical traits by Pearson correlation, with a Student asymptotic *P* value test for significance.

### Bayesian Inference in GRN and KD Analysis

Bayesian network inference was performed as described previously,^17^ in brief, the R rcausal package (v0.99.0). The Fast Greedy Equivalence Search (max degrees, 100) for continuous data algorithm was used to generate the Bayesian network on the WGCNA-generated modules. This provided directionality to the relationships (edges) in the network. KD analysis was performed as integrated in the Mergeomics package (v1.10.0), number of permutations was set to 20. KDs were considered significant if false discovery rate was <0.1.

### Bulk RNAseq of BCA Lesion Microdissections From Apoe^−/−^ Mice

Total RNA was isolated using RNeasy kit (Qiagen 74106) from the micro-dissected BCA lesions of Cdh5 Cre ERT2 R26R-eYFP Apoe*^−/−^* mice fed a high-fat WD for 18 or 30 weeks. The RNA library was prepared according to manufacturer instructions with rRNA reduction with an average RNA integrity number of 6.6. A total of 17 BCA RNA samples were isolated from mice (male, 18 weeks=5; male, 30 weeks=3; female, 18 weeks=5; and female, 30 weeks=4). A total of 4 samples were discarded due to low yield of RNA libraries, resulting in a total of 13 RNA samples for analysis (male, 18 weeks=5; male, 30 weeks=2; female, 18 weeks=3; and female, 30 weeks=3). RNA libraries were provided to the company Novogene, Inc (United States) for bulk RNAseq. Libraries were sequenced with the Illumina short-read sequencing (Illumina HiSeq v4; 100 bp and 25 million paired-end reads). Raw reads were QCed to exclude reads containing adapters, reads containing ambiguous calls (N) >10%, and reads where 50% of bases had a Qscore <5. QCed reads were mapped to the *Mus musculus* mice reference genome using the STAR software.^34^ Reads per transcript were extracted from the STAR output and converted to fragments per kilobase of transcript sequence per millions base pairs sequenced. A total of 54 532 transcripts were obtained of which 21 116 transcripts had an average expression >1 read per sample and were used for differential gene expression. Read counts were normalized and differential gene expression between groups was performed using DESeq2 in R.^35^ Reactome gene enrichment analysis for genes with significant up- or downregulation was performed.

### Single-Cell RNAseq of Human Carotid Plaques

Single-cell RNAseq was sequences following previously published protocols.^36,37^ In brief, atherosclerotic lesions were collected from 20 female and 26 male patients undergoing a carotid endarterectomy procedure. All pathological tissue was included in the Athero-Express Biobank Study biobank (AE, www.atheroexpress.nl) at the University Medical Center Utrecht.^21^ After sequencing, data were processed in an R 3.5 and 4.0 environment using Seurat, version 3.2.2.^38^ Mitochondrial genes were excluded, and doublets were omitted by gating for unique reads per cell (between 400 and 10 000) and total reads per cell (between 700 and 30 000). Data were corrected for sequencing batches using the function SCTransform. Clusters were created with 20 principal components, this number of components had significant proportion test compared with the proportion of features expected under a uniform distribution of *P* values. This was assessed using the JackStraw analysis in the Seurat package.^39^ Furthermore, the resolution of 0.8 was used for clustering as a low value that represented meaningful biological clustering to avoid overfitting. We performed a sensitivity analysis to evaluate the effect of different clustering parameters to the cell-type groups in this study, determining the effect to be marginal (Table S2; Figure S2). Multiple iterations of clustering were used to determine optimal clustering parameters. Population identities were assigned by evaluating gene expression per individual cell clusters. Subpopulations of SMCs were determined by isolating the SMC and EC populations from the complete population of cells mentioned above and reassigned clusters. Clusters were created with 10 principal components at resolution 1.4. New subtypes of SMC and EC identities were assigned by evaluating the DEGs per cell clusters using enrichment analysis in enrichR (v. 3.0).^40^ Module scores for the different sets of KD genes were generated using the addModuleScore function in Seurat,^38^ which estimates a proxy of expression for all the genes in the provided set.

## RESULTS

### Patient Population

**Figure 1.**
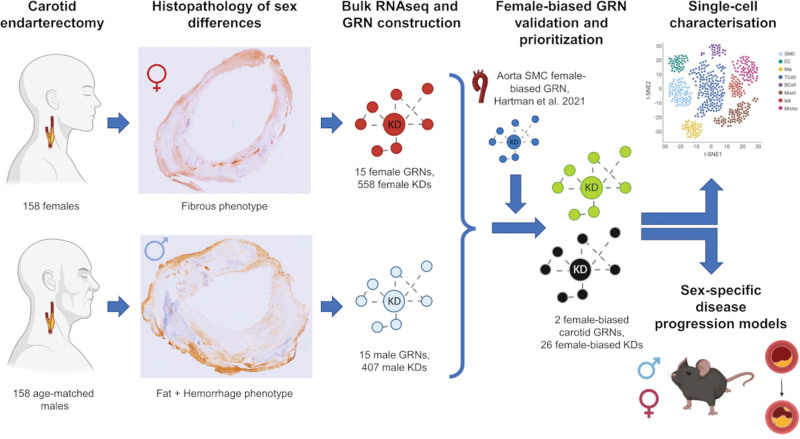
**Central illustration of the study.** Cartoon images created using BioRender. GRN indicates gene regulatory network; and KD, key driver.

To create robust and reliable female-biased GRNs of atherosclerosis, we used a systems biology approach in an equally powered cohort of atherosclerotic plaques from women and men.^17^ We selected all women with carotid plaque RNAseq data (n=158) and age-matched these with 158 men from the Athero-Express carotid endarterectomy biobank^21,41^ (Supplemental Methods; Figure 1). Differences in the male and female populations with respect to their risk factor profiles included significant higher HDL (high-density lipoprotein) in women (1.3 versus 1.0 mmol/L; *P*<0.001), a higher proportion of current smoking in women (46% versus 32% in men; *P*<0.001), and higher prevalence of alcohol consumption in men (72% versus 48%; *P<*0.001; Table S3). A higher prevalence of CAD history was found in men compared with women (37.3% versus 24.7%; *P=*0.021) as well as significantly higher use of anticoagulants in men compared with women (17% versus 7%; *P*=0.007). As reported previously,^8^ female plaques were more fibrous—highlighted by a high content of SMCs and low content of macrophages (MΦ). Conversely, male plaques showed a phenotype with a higher prevalence of plaque hemorrhage and fat content (Table S3).

### Construction of Sex-Specific GRNs in Carotid Atherosclerotic Plaques

**Figure 2.**
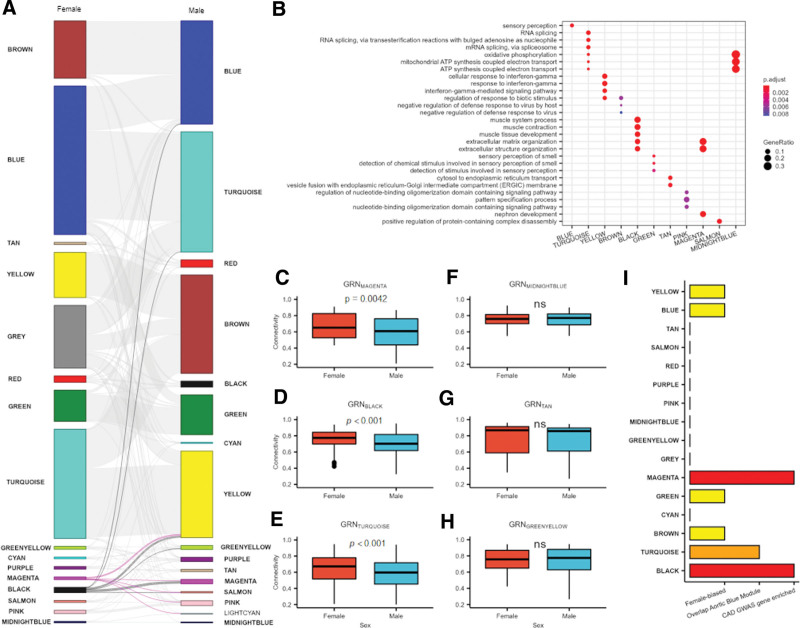
**Generating sex-stratified gene regulatory networks (GRNs) and prioritization of female-biased GRN in athero-express carotid tissue. A**, Representation of the gene regulatory networks in men and women. Highlighted are the corresponding male networks for the 2 female prioritized GRNs (GRN_MAGENTA_ and GRN_BLACK_). **B**, Gene enrichment for female networks (see Figure S2 for male-defined networks). **C** through **H**, Sex-stratified connectivity for 6 of the GRNs (magenta, black, turquoise, tan, midnight blue, and greenyellow). **C** through **E**, Female-biased GRNs (GRN_MAGENTA_, GRN_BLACK_, and GRN_TURQUOISE_; higher connectivity in women than men), while **F** through **H** represent nonbiased networks; the black line represents the line of identity. **I**, Prioritization of GRNs based on evidence of female bias, overlap with aortic STARNET blue module and enrichment for coronary artery disease genome-wide association study genes. Colors in **I** represent prioritization criteria met for each module: yellow, meets 1 criterion; orange, meets 2 criteria; red, meets all 3 criteria. Comparison of eigengene (**C–H**) was performed using a 2-tailed unpaired Student *t* test.

De novo unbiased coexpression networks were constructed in a sex-stratified manner using gene expression data from advanced plaques, which revealed 16 modules in the male group and 15 modules in the female group (Figure 2A; Figure S3; Table S4). To identify genes that drive the expression of the GRNs, we used Bayesian Network Inference and determined KD genes as previously described.^17^ A total of 558 KD genes were identified in female networks (Table S4), and 407 KD genes were identified in male networks at false discovery rate <0.1 (Table S5). The median size in female networks was 139 genes (interquartile range, 80–1595). In women, we identified 2 GRNs (GRN_MAGENTA_ and GRN_BLACK_) enriched for SMC-related processes. The GRN_BLACK_ was enriched for muscle system process and contraction as well as ECM and structure organization. GRN_MAGENTA_ was predominantly enriched for ECM and structure organization (Figure 2B; Table S6). The enrichment for SMC-like processes was highlighted by the top KD genes for the individual networks. The top KD genes for GRN_MAGENTA_ were *MFGE8*, *VCAN* (versican), and *NFIB* (nuclear factor I B). The top KD genes for GRN_BLACK_ were *BGN* (biglycan), *ACTA2* (actin alpha2, smooth muscle), and *DSTN* (destrin, actin depolymerizing factor; Table S3). Furthermore, we identified a large female network (1400 genes) enriched for immune processes (GRN_YELLOW_) and 1 network enriched for oxidative phosphorylation and ATP synthesis (GRN_MIDNIGHTBLUE_). The last network that we highlight was GRN_TURQUOISE_ (3358 genes) and was enriched for RNA splicing and transcription processes (Figure 2A; Table S6).

In men, the median GRN size was 149 genes (interquartile range, 65.5–1704.8). We identified 2 large networks representing immune processes (GRN_BROWN_, 3023 genes) and RNA splicing and transcription (GRN_YELLOW_, 2649 genes; Figure S4; Table S7). In contrast to what we found in women, we identified only 1 network representing SMC-like processes (GRN_MAGENTA_), which included *DSTN*, *ACTA2*, and *CALD1* (caldesmon 1) as top KD genes. This was also seen in the larger networks (>1000 genes) where networks with similar enrichment differed in size in the opposite sex (Figure 2A; Figure S3).

### Prioritization of Female-Biased Networks in Carotid Atherosclerotic Plaques

Next, we prioritized our female networks based on the following: (1) Female bias in network activity (based on network connectivity^16,17^ differences between sexes), (2) significant overlap in genes with the previously identified SMC female-biased network,^17^ and (3) colocalization of genes in the network with known CAD genome-wide association studies loci.^42,43^

Seven out of 15 of the networks identified in female plaques (47%) presented with sex bias defined as: a significantly higher connectivity in female plaques compared with male plaques (Figure 2C through 2H; Table S8). The gene composition of 3 of the 7 female-biased networks had significant overlap with the previously reported female network expressed in SMCs^17^ (*P*<0.04; Figure S5A; Table S9). We identified a total of 409 genes (38 KD genes) in these 3 female plaque networks that overlapped with the 775 genes previously observed (63 KD genes).^17^ The 3 overlapping networks in female plaques were enriched for genes involved in ECM organization, collagen organization, and angiogenesis (GRN_MAGENTA_) as well as genes involved in muscle contraction, muscle system process, and extracellular organization for (GRN_BLACK_). GRN_TURQUOISE_ was enriched for RNA processing–related processes (all *P*<0.001, Figure 2B; Tables S6 and S7).

**Figure 3.**
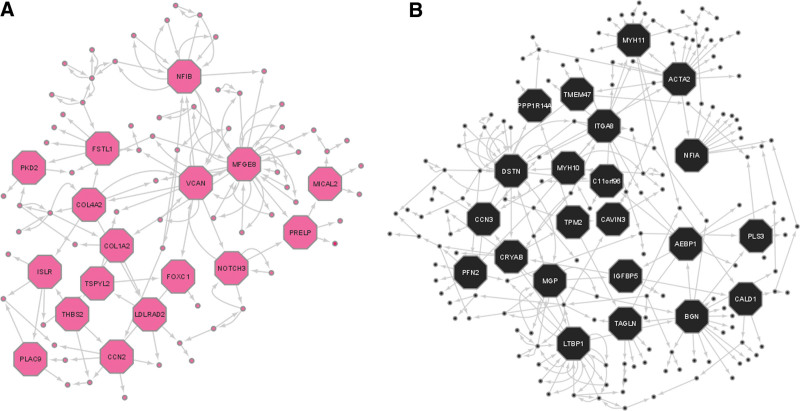
**Two female-biased gene regulatory networks (GRNs) identified in carotid plaques and prioritized based on smooth muscle cell biology and enrichment for coronary artery disease genome-wide association study loci. A**, GRN_MAGENTA_: 82 genes, 17 key driver (KD) genes (octagonal nodes of the network, false discovery rate [FDR] <0.1). **B**, GRN_BLACK_: 171 genes, 22 KD genes (octagonal nodes of the network, FDR<0.1). The directionality of the edges is obtained from Bayesian network inference in each network (Methods). This figure is represented as a summary highlighting the 2 main networks described in this article, please refer to https://ediezben.github.io/sex_diff_AE_networks for a detailed view of the networks.

Genome-wide association studies have shed light on mechanisms of CAD.^42,43^ Therefore, we studied which networks were enriched with genes within loci known to associate with CAD.^42,43^ Two out of the 3 female-biased networks (GRN_MAGENTA_ and GRN_BLACK_) were enriched for genes within CAD loci (*P<*0.001, Table S9; Figure S5B). Using this prioritization approach, GRN_MAGENTA_ and GRN_BLACK_ were prioritized for downstream analysis (Figure 2I; Figure 3).

We identified 2 male networks, which had a near-significant sex bias when compared with the equivalent networks in women (Table S11). Gene enrichment in this network highlighted signaling and signal transduction as the main pathways for the male GRN_PINK_ and cell-cell junction and neuronal development for GRN_PURPLE_ (Figure S4; Table S7).

### Prioritized Female-Biased GRNs Represent 2 SMCs Phenotypes That Modulate Plaque Stability in Women and May Play a Role in Modulating EC Transitions

**Figure 4.**
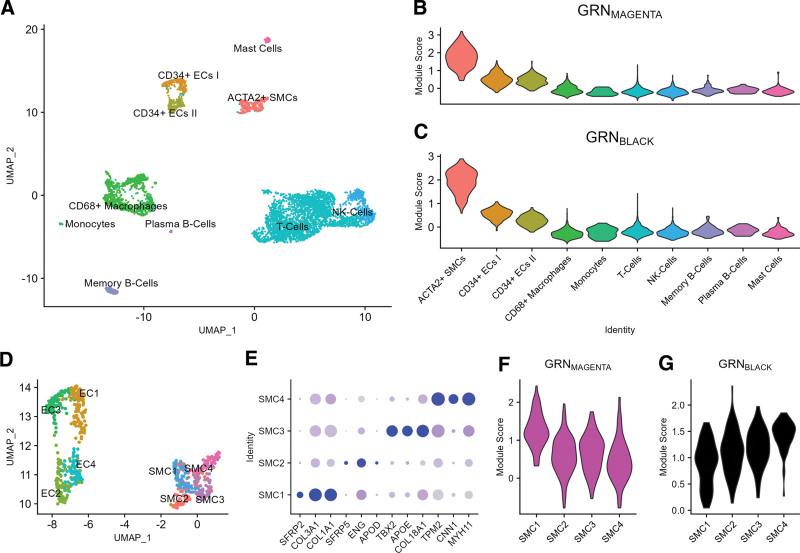
**Female-biased gene regulatory networks (GRNs) are expressed in different phenotypes of smooth muscle cells (SMCs) including a myofibroblast-like phenotype. A**, UMAP plot of 4948 single cells from carotid plaque (46 patients, 26 men and 20 women). **B**, Module score (Supplemental Methods) expression of GRN_MAGENTA_ key driver (KD) genes in carotid plaque cell types. **C**, Module score expression of GRN_BLACK_ KD genes in carotid plaque cell types. **D**, Zoom-in UMAP plot of 672 smooth muscle and endothelial single cells and their clusters from carotid plaque (46 patients, 26 men and 20 women). **E**, Top 3 DEGs for each SMC subtype, circle size represents percentage of cells expressing the gene, and blue intensity represents the normalized expression level. **F**, Module score expression of GRN_MAGENTA_ KD genes in SMC subtypes. **G**, Module score expression of GRN_BLACK_ KD genes in SMC subtypes. APOD indicates apolipoprotein D; APOE, apolipoprotein E; CNN1, calponin 1; COL1A1, collagen type I alpha 1 chain; COL3A1, collagen type III alpha 1 chain; COL18A1, collagen type XVIII alpha 1 chain; DEG, differentially expressed gene; EC, endothelial cell; ENG, endoglin; MYH11, myosin heavy chain 11; SFRP2, secreted frizzled related protein 2; SFRP5, secreted frizzled related protein 5; TBX2, T-box transcription factor 2; TPM2, tropomyosin 2; and UMAP, Uniform Manifold Approximation and Projection.

To study these 2 prioritized female GRNs in more detail, we focused on their cellular origin. We used extended single-cell RNA sequencing (scRNAseq) data from carotid plaques of 46 patients (20 women and 26 men, Figure 4A)^36,37^ to study the cell-type expression of KD genes from GRN_MAGENTA_ and GRN_BLACK_. The scRNAseq identified a total of 20 cell clusters,^37^ which were merged into 10 cell-type clusters. The expression of KD genes from both networks was highest in SMCs as measured through a module score (Supplemental Methods). However, we also identified high expression in both clusters of ECs (Figure 4B and 4C). More specifically, genes from GRN_MAGENTA_ were equally expressed in both EC clusters (Figure 4B) while GRN_BLACK_ was higher expressed in EC1 (Figure 4C).

Next, we examined the different cell-type clusters in SMCs (Figure 4D and 4E; Table S12). GRN_MAGENTA_ was higher expressed in *KLF4*^+^ SMC1 subtype compared with the other SMC subtypes (Figure 4F; Figure S6A). This SMC subtype matches previously reported descriptions of phenotypically modulated myofibroblasts^44^ expressing both classic SMCs markers *ACTA2*, *TAGLN* (transgelin), and *MYL9* (myosin light chain 9), and fibroblast markers such as *FBLN1* (fibulin 1), *DCN* (decorin), and *SFRP2* (secreted frizzled related protein 2; Figure S7; Table S12). Expression of GRN_MAGENTA_ by myofibroblast-like cells was also validated in additional scRNAseq data sets from carotid endarterectomy^45^ and from coronary arteries from transplant patients.^46^ Expression of the KD genes from GRN_MAGENTA_ was the highest in phenotypically modulated fibrochondrocytes in the carotid data set^45^ (Figure S8A and S8B) and in modulated TNFRSF11B^+^ (TNF receptor superfamily member 11b) fibromyocytes (Figure S9) in the coronary tissue^46^ (Figure S8D and S8E). Furthermore, the sex ping of the cells in these data sets using sex chromosome gene expression revealed that the proportion of *TNFRSF11B^+^* cells over other SMC-like cells was higher in women than in men in both cases (Figures S10 and S11). In contrast, GRN_BLACK_ was higher expressed in *TPM2^+^CNN1^+^MYH11^+^* (tropomyosion 2/calponin 1/myosin heavy chain 11) SMC4 contractile subtype compared with the other clusters of SMCs, with SMC1 presenting the lowest expression (Figure 4G). GRN_BLACK_ KD gene expression was the highest in canonical SMC clusters from the complementary scRNAseq data sets (Figures S8C, S8F, S9B, and S9D).

Modulated SMCs have been suggested to play an atheroprotective role in plaque progression^46^; however, they also give rise to other detrimental cellular states, such as osteogenic SMCs^19,47–49^ and foam cells.^50–53^ to study the 2 GRNs in relation to plaque phenotypes in women, we assessed plaque histology. The expression of GRN_MAGENTA_ compared with GRN_BLACK_ was higher in 82 out of 158 (52%) female plaques (Figure S12). These plaques were more vulnerable and atheromatous (plaque vulnerability index: 3.4 versus 2.8, *P*<0.001; atheromatous features: 70% versus 45%, *P*=0.005). We also observed a nonsignificant trend toward more intraplaque hemorrhage (present: 60% versus 44%, *P*=0.067) and less *ACTA2*^+^ SMCs content (rank-normalized SMC content=0.06 versus 0.37, *P*=0.074) in plaques with high expression of GRN_MAGENTA._ Furthermore, these female patients presented with severe symptoms (stroke symptoms: 33% versus 19%, *P*=0.074; Table 2).

**Table 2.**
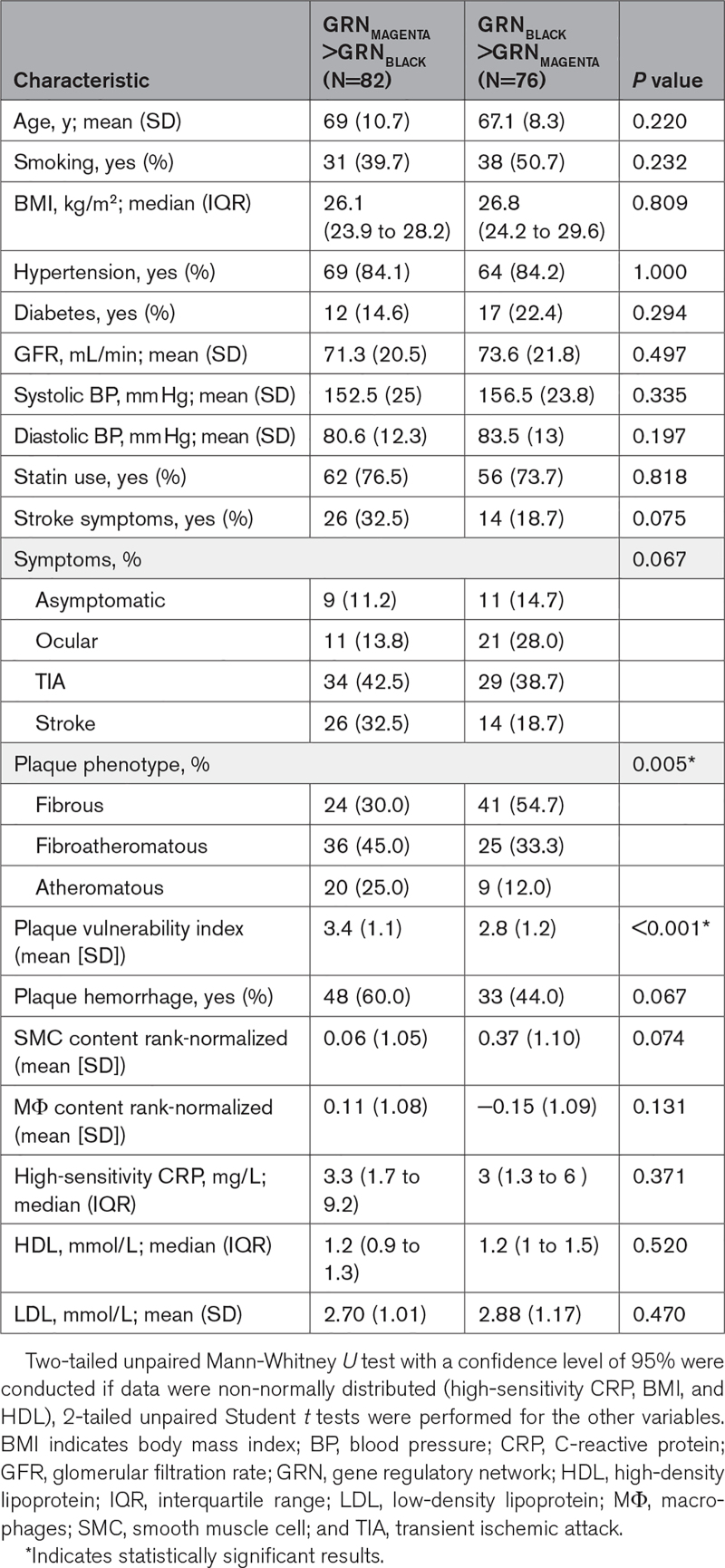
Clinical and Carotid Plaque Characteristics of 158 Female Patients With Different Expression of SMC-Related GRNs

### Female-Biased GRNs Are Expressed in ECs With Signs of EndMT

**Figure 5.**
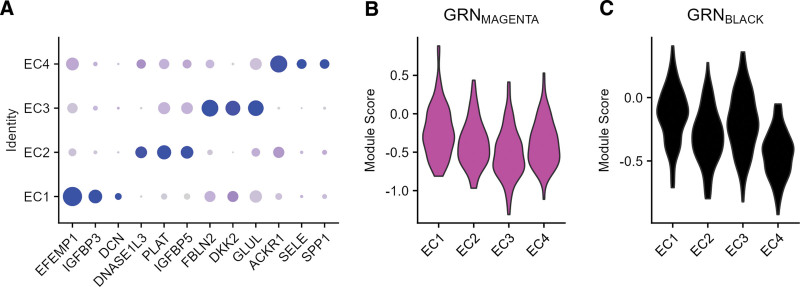
**Female-biased gene regulatory networks (GRNs) are expressed in endothelial cells (ECs) with strong signals of endothelial to mesenchymal transition: A**, Top 3 differential expressed genes for each EC subtype, circle size represents percentage of cells expressing the gene, and blue intensity represents the normalized expression level. **B**, Module score expression of GRN_MAGENTA_ key driver (KD) genes in EC subtypes. **C**, Module score expression of GRN_BLACK_ KD genes in EC subtypes. ACKR1 indicates atypical chemokine receptor 1 (Duffy blood group); DCN, decorin; DKK2, dickkopf WNT signaling pathway inhibitor 2; DNASE1L3, deoxyribonuclease 1 like 3; EFEMP1, EGF containing fibulin extracellular matrix protein 1; FBLN2, fibulin 2; GLUL, glutamate-ammonia ligase; IGFBP3, insulin-like growth factor binding protein 3; IGFBP5, insulin-like growth factor binding protein 5; PLAT, plasminogen activator, tissue type; SELE, selectin E; and SPP1, secreted phosphoprotein 1.

We previously identified EndMT as a main enrichment in the female-biased SMC GRN.^17^ This suggests a potential role of EndMT in driving sex differences in atherosclerosis. We, therefore, investigated the expression levels of GRN_MAGENTA_ and GRN_BLACK_ in EC subtypes (Figure 5D). Expression levels of both networks were the highest in EC1 compared with other EC subtypes, which represents a *SULF1*^+^ (sulfatase 1; Figure 5A) subtype (of cells that are *CD34*^+^) and are positive for *ACTA2*^+^ (Figure S6B). EC1 also counts *EFEMP1* (EGF containing fibulin extracellular matrix protein 1), *IGFBP3* (insulin-like growth factor binding protein 3), and *DCN* (Figure 5B and 5C; Table S12) among their top differentially expressed genes, which are fibroblast-like markers. In addition, we identified enrichment for *TGF*β** response, SMC proliferation, and endothelial to mesenchymal transition for EC1 (*P<*0.001 for GO term enrichment; Table S14).

### Expression of KD Female-Biased Genes Is Maintained in Female Apoe^−/−^ Mice During Disease Progression but Downregulated in Men

**Figure 6.**
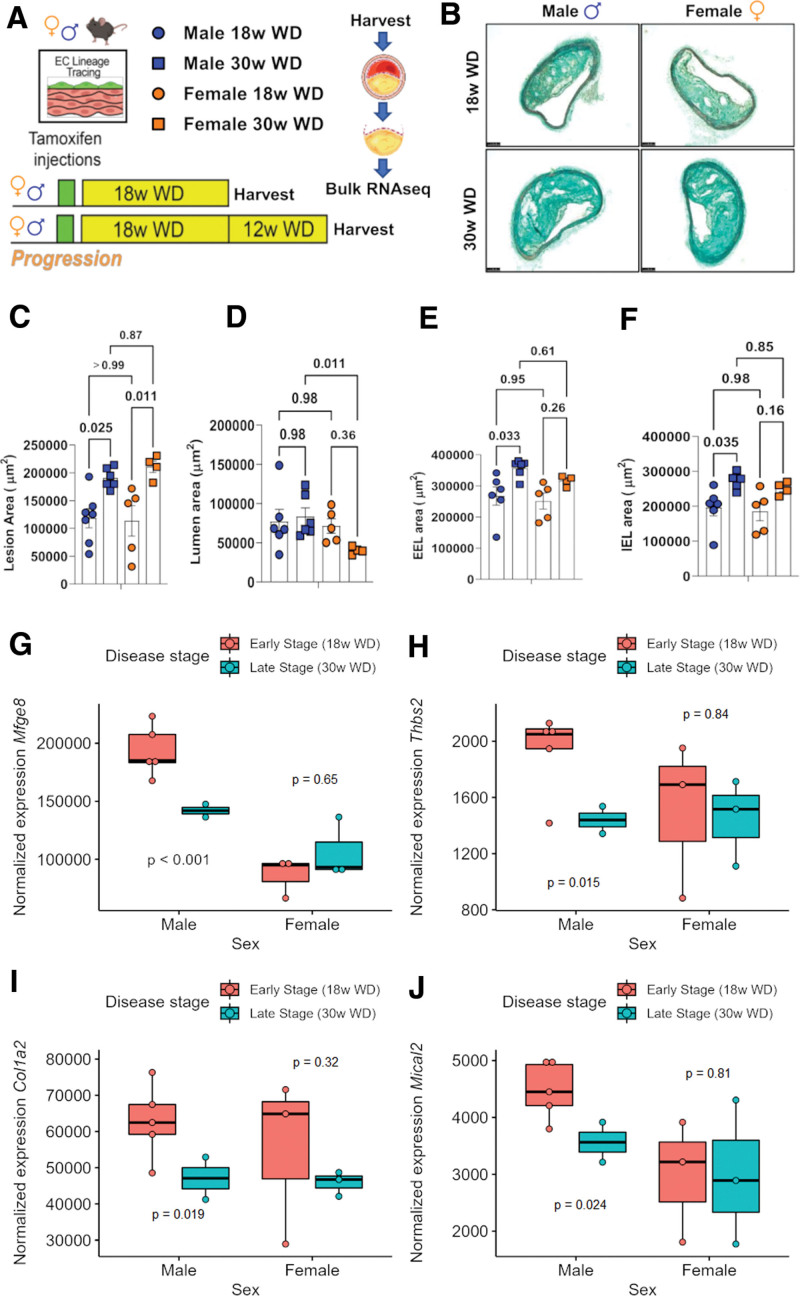
**Male-specific downregulation of key driver (KD) genes from GRN_MAGENTA_ is observed during atherosclerosis progression in brachiocephalic artery (BCA) lesions from Apoe*^−/−^* mice fed a Western diet (WD). A**, Diagram for experimental design in both male and female mice-fed WD for 18 and 30 weeks. **B**, Movat staining of representative lesions for male and female mice at 18 and 30 weeks of WD. **C**, Lession area of male and female mice at 18- and 30-week WD. **D**, Lumen area of male and female mice at 18- and 30-week WD. **E**, External elastica lamina (EEL) area of male and female mice at 18- and 30-week WD. **F**, Internal elastica lamina (IEL) area of male and female mice at 18- and 30-week WD. **G**, Normalized expression of *Mfge8* gene by sex and disease stage. **H**, Normalized expression of *Thbs2* gene by sex and disease stage. **I**, Normalized expression of *Col1a2* gene by sex and disease stage. **J**, Normalized expression of *Mical2* gene by sex and disease stage. *Cdh5-Cre ERT2 R26R-eYFP Apoe^−/−^* mice (species: *Mus musculus*, age: 6 weeks at the time of tamoxifen treatment, sex: 13 men and 9 women, strain: C57BL/6J, source: The Jackson Laboratory) were used in this study for morphometry analysis. A total of 17 BCA RNA samples were isolated from mice (male, 18 weeks=5; male, 30 weeks=3; female, 18 weeks=5; and female, 30 weeks=4). A total of 4 samples were discarded due to low yield of RNA libraries, resulting in a total of 13 RNA samples for analysis (male, 18 weeks =5; male, 30 weeks=2; female, 18 weeks=3; and female, 30 weeks=3). Two-tailed unpaired Mann-Whitney *U* tests with a confidence level of 95% were conducted if data were non-normally distributed. For comparison of 2 groups of continuous variables with normal distribution and equal variances, 2-tailed unpaired Student *t* tests were performed with a confidence level of 95%.

To study the role of the 2 female GRNs in atherosclerosis progression, we used female and male Apoe*^−/−^* mice (n=9 and 13, respectively) that were fed a WD for 18 weeks (18-week WD, that is, early-stage atherosclerosis) or 30 weeks (30-week WD, that is, late-stage atherosclerosis; Figure 6A). Lesion size was assessed using Movat staining. A significant increase in lesion size was observed both in men and women in late stages when compared with early atherosclerosis stages (*P<*0.02; Figure 6B and 6C), and no significant difference was observed in lumen size (Figure 6D). Brachiocephalic arteries were successfully harvested from a total of 17 mice from an independent experiment (women, 9; men, 8), and the lesion site was successfully laser micro-dissected and processed using bulk RNAseq (women, n=6; men, n=7; Supplemental Methods; Figure 6A). A total of 13 083 transcripts out of 21 116 mice transcripts recovered overlapped with the human genes used in our carotid study, including 75/82 (91%) and 160/171 (94%) of GRN_MAGENTA_ and GRN_BLACK_ genes, respectively. We performed sex-stratified differential gene expression analysis between conditions (late-stage versus early-stage atherosclerosis) and differential gene expression analysis between sexes (men versus women) at both disease stages (Tables S14 through S18). We identified a male-specific upregulation of calcification and osteogenic processes, translation, and RNA processing in late-stage atherosclerosis. Also, we observed a downregulation of ECM organization genes (Figure S13; Table S20). Among these, expression of the KD gene *Mfge8* (milk fat globule EGF and factor V/VIII domain containing) of GRN_MAGENTA_ was significantly downregulated in late-stage atherosclerosis in male mice, while expression was maintained in female plaques (Figure 6G). Other KD genes of GRN_MAGENTA_ (including *Col1a2* [collagen, type I, alpha 2], *Thbs2* [thrombospondin 2], and *Mical2* [microtubule-associated monooxygenase, calponin and LIM domain containing 2]) showed a marginally significant trend in the same direction (Figure 6H through 6J). These results suggest a male-specific decrease in the expression of the *Mfge8* gene during disease progression while this gene expression is maintained in female mice plaques.

## DISCUSSION

In this study, we identify 2 female-biased GRNs that are highly expressed in SMCs and ECs, associate with plaque composition and vulnerability, and seem to remain stable in expression during atherosclerosis progression in female mice, and not in male mice. Our data show that female atherosclerosis is characterized by phenotypic modulation of SMCs. We show high expression of these GRNs in vulnerable fibrous plaques rich in ECM-producing myofibroblast-like SMCs. Furthermore, we highlight the importance of sex stratification in atherosclerosis research to find new mechanisms of disease progression.

At the gene expression level, we recently uncovered sex differences in gene connectivity in atherosclerotic tissue, which pointed to SMC and ECs as main players in female atherosclerosis.^17^ In this study, we substantiate that this dominant female SMC and EC biology is not only important for arterial tissue from patients with CAD but also for carotid plaques from patients with stroke. Our mouse data support the idea that there are intrinsic differences in male and female atherosclerosis pathology progression, as confounding by different risk factor distributions does not play a role in our in vivo experimental setting. As sex hormones and chromosomes determine biological sex, it may be that the XX sex chromosome complement accelerates atherosclerosis in women, as was found in the murine Four Core Genotype model.^54^ Besides the potential role of the sex chromosome complement, estrogen receptor signaling was also previously found in enrichment analyses of female atherosclerotic plaques of women of an average age of 70 years, long after menopause.^17^ This may imply that estrogen may still be an important factor to consider in postmenopausal women in vascular tissues and suggests long-term epigenetic effects.

In line with our previous work, female-biased GRNs in carotid plaques pointed to SMC biology. However, we now have evidence that the previously identified female SMC GRN^17^ might encompass larger diversity at late stages of atherosclerosis development, with 2 main networks identified in symptomatic atherosclerotic plaques. GRN_MAGENTA_ overlaps in expression with modulated SMCs and points toward a more vulnerable plaque phenotype including a higher prevalence of atheroma, which reflects the dual role that modulated SMCs might play in lesion stability depending on their end-stage fate. It is worth noting other factors, such as the higher inflammation observed in male plaques compared with females, as well as known differences in vessel size that may also shape the fate of SMCs, and potentially ECs, in the vessel wall. Interestingly, between different vascular beds, mainly aortic^17^ and carotid, the molecular mechanisms that are sex-biased point to similar biological processes and cellular compartments. This corresponds to the consistent sex differences seen in plaque histology from different vascular beds. We have previously described a higher prevalence of lipid core and plaque hemorrhage in both carotid and iliofemoral male plaques compared with female plaques,^3^ and a higher prevalence of fibrous plaques in women. These differences in mechanisms may have consequences for emerging antifibrotic or anti-inflammatory treatments,^55,56^ which may differ in efficacy by sex.

In this study, we collected plaque samples in the last stage of atherosclerosis, which represents the sum of accumulated disease progression over time. While sex differences in atherosclerotic plaques were described to be especially pronounced at younger ages (<50 years),^57^ the female plaques in our cohort were consistently more fibrous compared with male plaques.^3,8^

In-depth characterization of the networks’ coexpression patterns in scRNAseq from carotid plaques highlighted that GRN_MAGENTA_ is highly expressed in a myofibroblast-like *KLF4*^+^ SMC subtype, and GRN_BLACK_ is mainly expressed in contractile SMCs. In line with this, previous studies have identified female KD genes such as *FN1* to be modulated by *KLF4*,^17^ which is the main transcription factor modulating synthetic SMCs, which represents an intermediate state that precedes the myofibroblast phenotype in several models.^49^ Our results further prove the importance of SMC plasticity in female atherosclerosis.

We have also identified that KDs of the GRN_MAGENTA_ are highly expressed in a subtype of ECs with biological signatures pointing to SMC proliferation and EndMT. This combined with the high expression of *ACTA2*^+^ in this same subtype suggests that this network may also play a role in EndMT and may contribute to plaque formation in women. However, studying the exact contribution of the SMC-driven and EC-driven network expression and their potential interactions would require more in-depth research. We postulate that advanced experimental settings such as scRNAseq data from lineage-traced mice in which the origin of the cells expressing these networks can be determined are an important next step to understand if mechanisms that lead to these modulated cell types are sex specific. Furthermore, the activity of the networks may vary depending on the region of the plaque and the stage of atherosclerosis. Future studies are needed to identify the spatial expression of key genes in the plaques and their expression over time.

It is worth noting that ECM proteins, which are mainstays of both female-biased carotid GRNs highlighted in this study, have been identified as key players within other disease mechanisms that present a marked sex bias, such as heart failure with preserved ejection fraction^58^ and myocardial remodeling.^59^ Furthermore, several of these genes have been shown to affect SMC phenotypic switching specifically, such as *FN1*,^60^ which is also a validated CAD genome-wide association studies gene.^61^ The gene identified as a KD in this study *MFGE8* is a CAD genome-wide association studies gene^62^ and has been suggested to interact with elastin^63^ and a common loss-of-function variant in a Finish population has been shown to associate with atheroprotection.^64^ Therefore, the importance of these ECM genes as mediators of sex-specific disease mechanisms should be further studied. The results of our study suggest that while ECM production (including the GRN_MAGENTA_ KD genes, *MFGE8*) is maintained in female mice’s atherosclerosis progression, it appears downregulated in male mice. One hypothesis is that cell types (ie, myofibroblasts) producing these ECM proteins are less prevalent in male mice plaques. Alternatively, the networks themselves could be less active in male cells as atherosclerosis progresses. It is also worth noting that while ECM-producing myofibroblasts are critical in the formation of the fibrous cap, they associate with vulnerable plaques in women. This suggests that the role of these ECM-producing myofibroblasts in advanced stages may also be detrimental, for example, by becoming a scaffold for calcification.^65^ to understand why these differences emerge, answer these questions, one would require high-quality male and female plaque scRNAseq data in both human and mice, preferably within the Four Core Genotypes mouse model to disentangle chromosome from gonadal sex.^54^

One of the main limitations of the Athero-Express study is the low number of female patients (30%). Therefore, to ensure equally powered studies in both male and female plaques, we downsampled the male cohort. This reduced the overall power of our study in men, which may have limited the capability to identify male-biased networks. Also, we used histological sections in this study that were scored using semiquantitative measures of stainings. This may be considered a limitation given the resolution in comparison to the highly complex gene expression patterns. However, these histological measures are widely used for plaque phenotyping and have good replication metrics.^3,21,23^ Another limitation is the lack of vascular control tissue in the Athero-Express biobank. However, we previously^17^ used healthy tissue from mammary arteries to select the disease-relevant networks. Given the large overlap between the 2 networks described here and the previous one, we assume that these networks are mainly active in atherosclerotic plaques. Another limitation is that we used network connectivity (the strength of coexpression between genes) as a proxy for network activity. This approach considers the strength of the interaction between nodes as the primary measure of activity. We choose this method to compare our networks to previous studies.^17^ However, other methods that estimate network connectivity using the number of edges between nodes could have been used. We also acknowledge the fact that tamoxifen, which is used in the EC-lineage-tracing mice, is an estrogen receptor modulator that has been shown to increase cardiovascular risk in women under long-term treatment for breast cancer^66^; however, the dose and duration of treatment (a daily injection of 1 mg during 10 days at the age of 6 weeks) is thought not to have a meaningful impact on atherosclerotic disease development over the next 30 weeks as effects of tamoxifen are observed over periods of years in humans.^66^ Furthermore, in this study, mice used were 6 weeks at the start of the experiment, and while our human studies are performed on advanced lesions, the atherosclerosis observed in mice is progressing and shows a similar trajectory of differences between men and women as observed in humans (ie, higher ECM in women than men).

## CONCLUSIONS

Female atherosclerosis is characterized by GRNs that are active in fibrous vulnerable plaques rich in myofibroblast-like SMCs. This study sheds light on sex-specific networks and their expression during atherosclerosis disease progression. We identify *MFGE8* as a potentially important gene involved in SMC plasticity in female atherosclerosis. Our results highlight the importance of sex-stratified studies in atherosclerosis to understand the mechanism of symptomatic fibrous plaques.

## ARTICLE INFORMATION

### Sources of Funding

This work has been partly funded with support from the UCARE Horizon 2020 European Research Council Consolidator Grant (ID: 866478) to H.M. den Ruijter and the Leducq Foundation Transatlantic Network of Excellence (AtheroGEN) to H.M. den Ruijter and M. Civelek. This work was supported by National Institutes of Health grants R01 HL156849, R01 HL136314, and R01 HL141425 to G.K. Owens, Leducq Foundation Transatlantic Network of Excellence (PlaqOmics) to G.K. Owens and G. Pasterkamp, and PlaqOmics Young Investigator Award to S. Karnewar, R. Aherrahrou, T. Reinberger, and E. Diez Benavente.

### Disclosures

None.

### Supplemental Material

Tables S1–S22

Figures S1–S13

Major Resources Table

## Supplementary Material
