# Supplementary material for: Female Gene Networks Are Expressed in Myofibroblast-Like Smooth Muscle Cells in Vulnerable Atherosclerotic Plaques
Source: Arterioscler Thromb Vasc Biol. 2023 Aug 17;43(10):1836–50. doi: 10.1161/ATVBAHA.123.319325 (PMC10521798; doi:10.1161/ATVBAHA.123.319325)
Supplement: Supplementary file 1 [file atv-43-1836-s001.pdf]

Supplementary Information

Female gene networks are expressed in myofibroblast-like smooth muscle cells in vulnerable atherosclerotic plaques.

Short title: Vulnerable atherosclerotic plaques in females

Ernest Diez Benavente [1], Santosh Karnewar [2], Michele Buono [1], Eloi Mili [1], Robin J. G. Hartman [1], Daniek Kapteijn [1], Lotte Slenders [3], Mark Daniels [1], Redouane Aherrahrou [4, 5, 6], Tobias Reinberger [5], Barend M. Mol [7], Gert J. de Borst [7], Dominique P. V. de Kleijn [7], Koen H. M. Prange [8], Marie A. C. Depuydt [8], Menno P. J. de Winther [8], Johan Kuiper [8], Johan L. M. Björkegren [9, 10], Jeanette Erdmann [5], Mete Civelek [4, 11], Michal Mokry [3], Gary K Owens [2], Gerard Pasterkamp [3], Hester M. den Ruijter [1, *]

[1] Laboratory of Experimental Cardiology, University Medical Center Utrecht, Utrecht University, the Netherlands

[2] Robert M. Berne Cardiovascular Research Center, University of Virginia, Charlottesville, VA, USA.

[3] Central Diagnostic Laboratory, University Medical Center Utrecht, Utrecht University, Utrecht, The Netherlands

[4] Center for Public Health Genomics, University of Virginia, Charlottesville, VA, USA.

[5] Institute for Cardiogenetics, University of Lübeck, Lübeck, Germany

[6] A.I. Virtanen Institute for Molecular Sciences, University of Eastern Finland, Finland

[7] Department of Vascular Surgery, University Medical Centre Utrecht, Utrecht, Utrecht University, The Netherlands

[8] Division of BioTherapeutics, Leiden Academic Centre for Drug Research, Leiden University, Leiden, The Netherlands

[9] Department of Genetics and Genomic Sciences, Icahn School of Medicine at Mount Sinai, New York, NY, USA

[10] Integrated Cardio Metabolic Centre, Department of Medicine, Karolinska Institutet, Karolinska Universitetssjukhuset, Huddinge, Sweden

[11] Department of Biomedical Engineering, University of Virginia, Charlottesville, VA, USA

[*] Corresponding author:

Hester M. den Ruijter, PhD
Division of Heart & Lungs
Department of Cardiology
Laboratory of Experimental Cardiology
H.M.denruijter-2@umcutrecht.nl

Postal address: Huispostnummer G03.550
P.O. Box 85500
3508 GA Utrecht
The Netherlands

Supplementary Figures

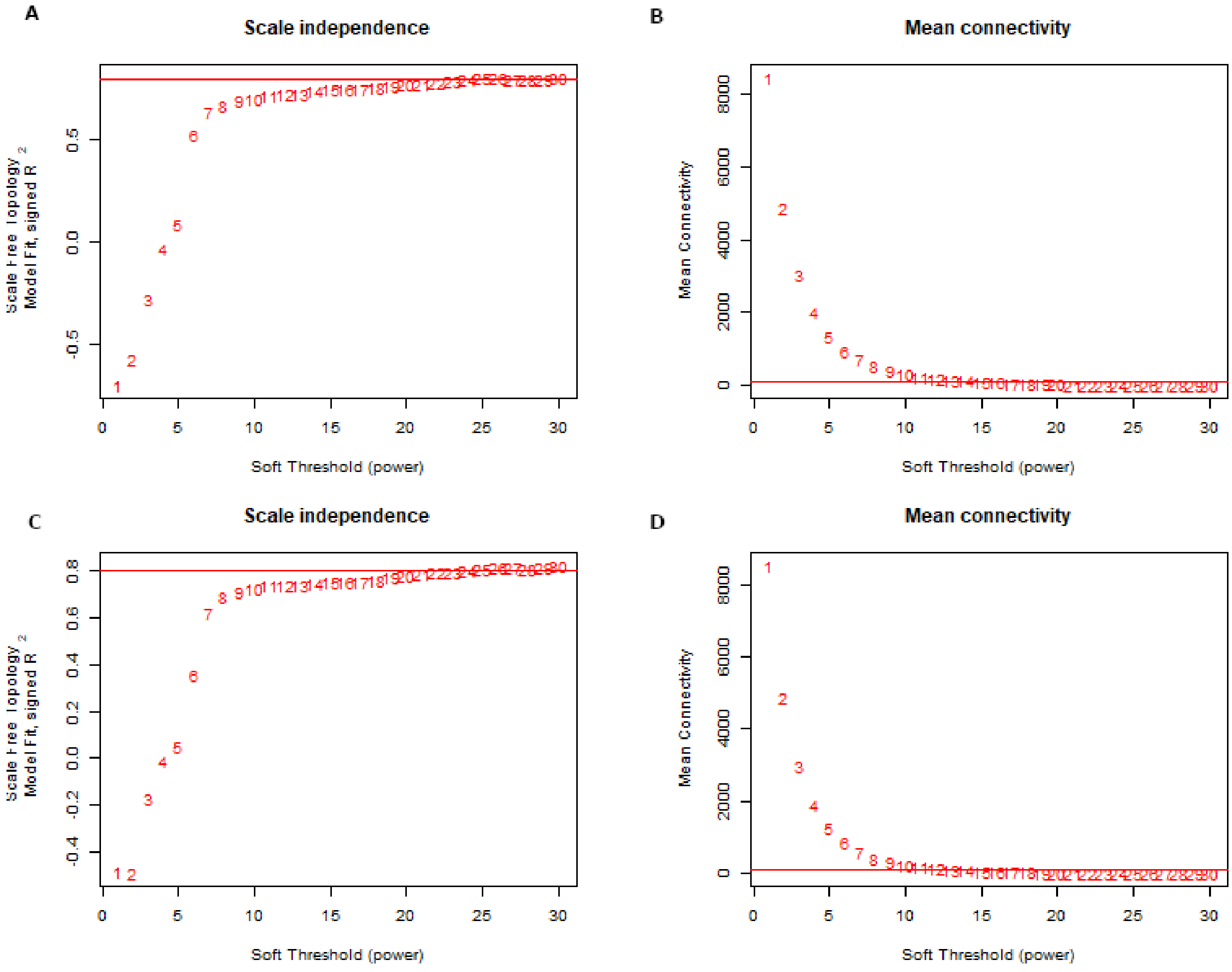

Figure S1. Parametrization of soft threshold selection for WGCNA network creation: A. Scale free topology Model Fit for different soft-thresholds in the female plaque dataset. B. Mean connectivity for different soft-thresholds in the female plaque dataset. C. Scale free topology Model Fit for different soft-thresholds in the female plaque dataset. D. Mean connectivity for different soft-thresholds in the female plaque dataset.

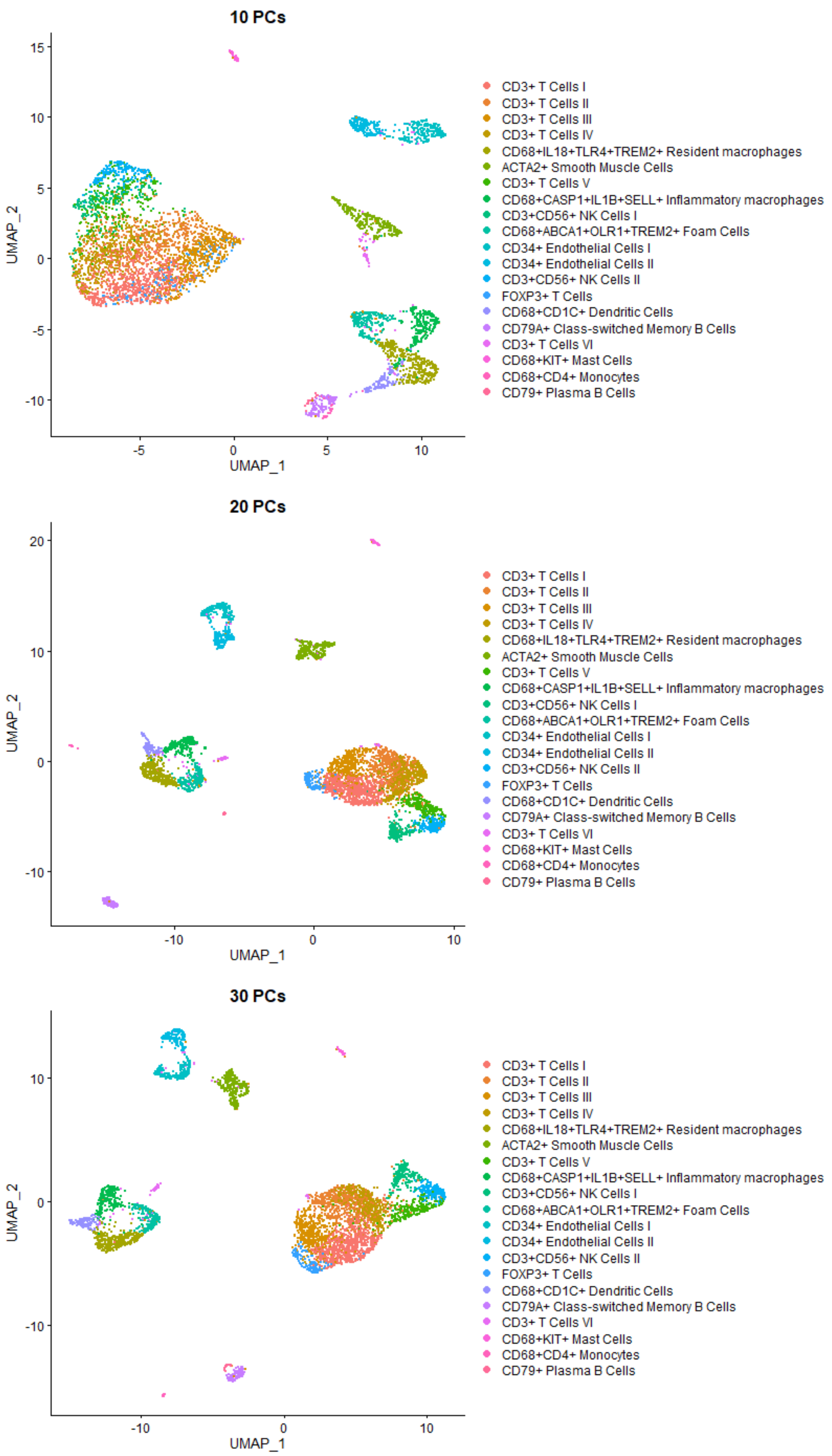

Figure S2. UMAP plots for comparison of clustering patterns based on the use of different set of PCs (10 PCs top, 20 PCs middle and 30 PCs bottom). *Cells are colored based on the clustering results using 20PCs and resolution 0.8 used in this study for comparison.*

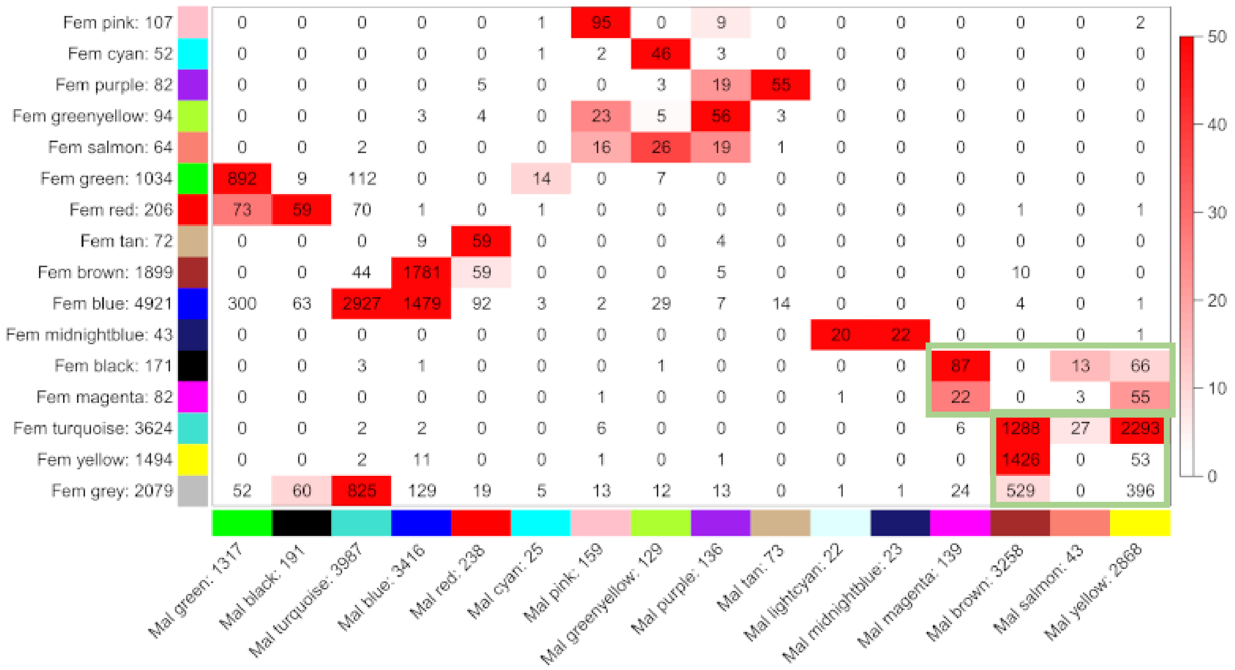

Figure S3. Comparison of female defined GRNs genes and male defined GRNs genes in atherosclerotic plaques from 158 females and 158 age-matched males. *The numbers represent the genes overlapping between the female and male modules. Boxes have been colored accoring to % ov overlap between modules. Fem = Females, Mal = Males.*

A

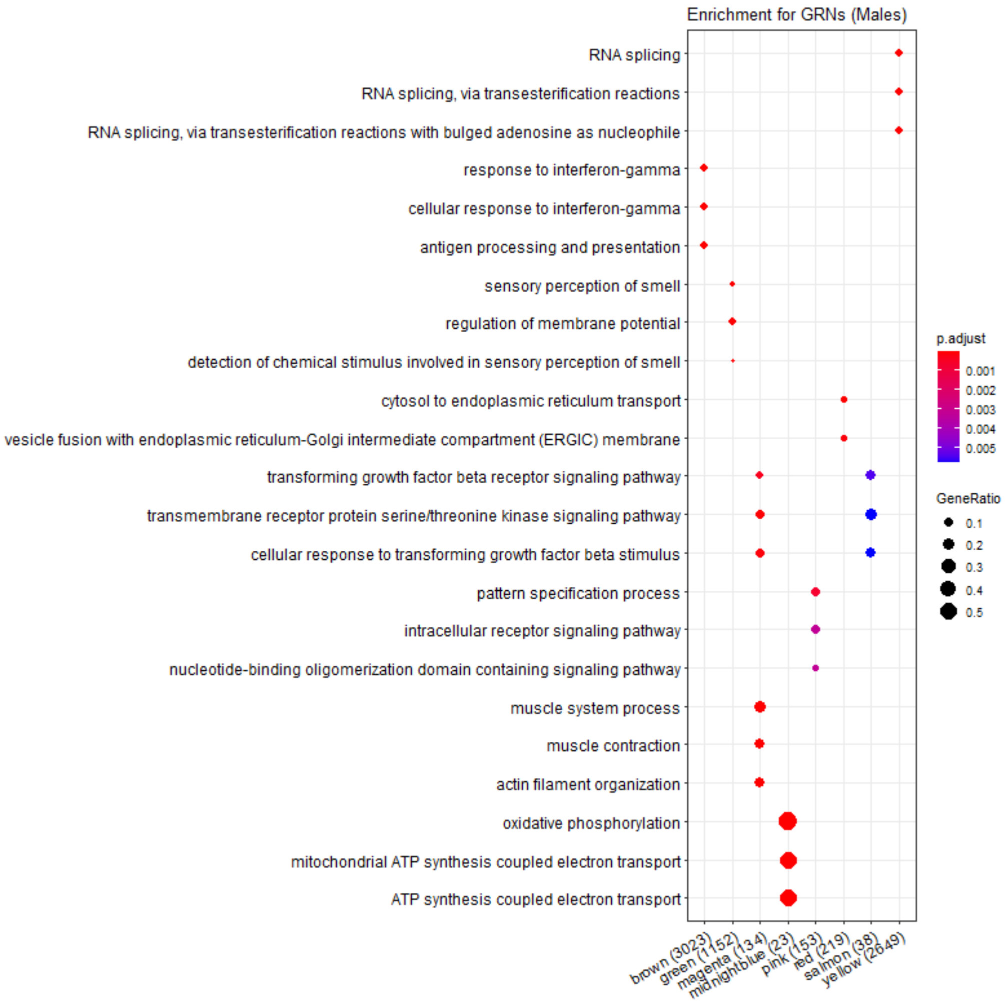

Figure S4. Top 3 enriched GO terms for male-defined plaque gene regulatory networks (GRNs).

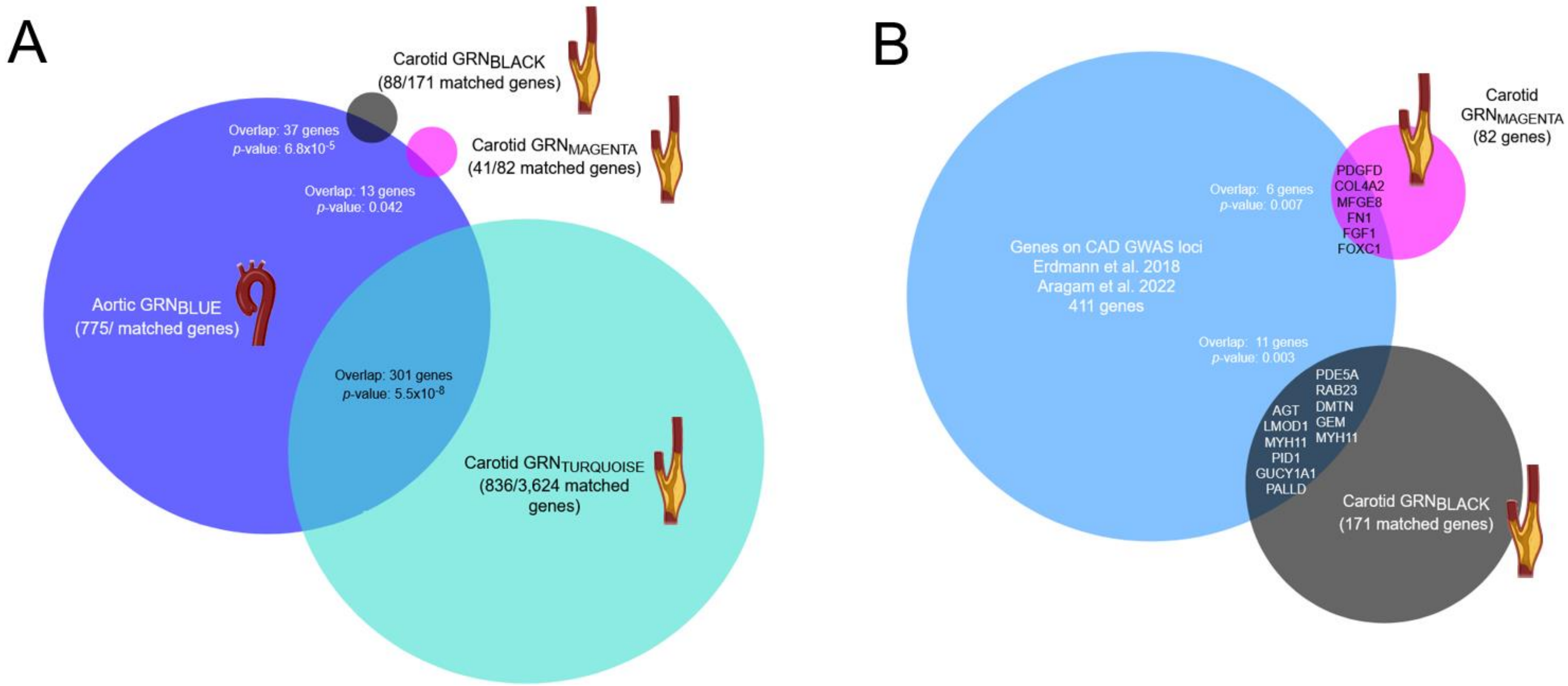

Figure S5. Prioritization criteria for GRN networks. A. Prioritization based on overlap with STARNET blue aortic GRN. B. Prioritization of GRNs using enrichment for genes in CAD GWAS loci.

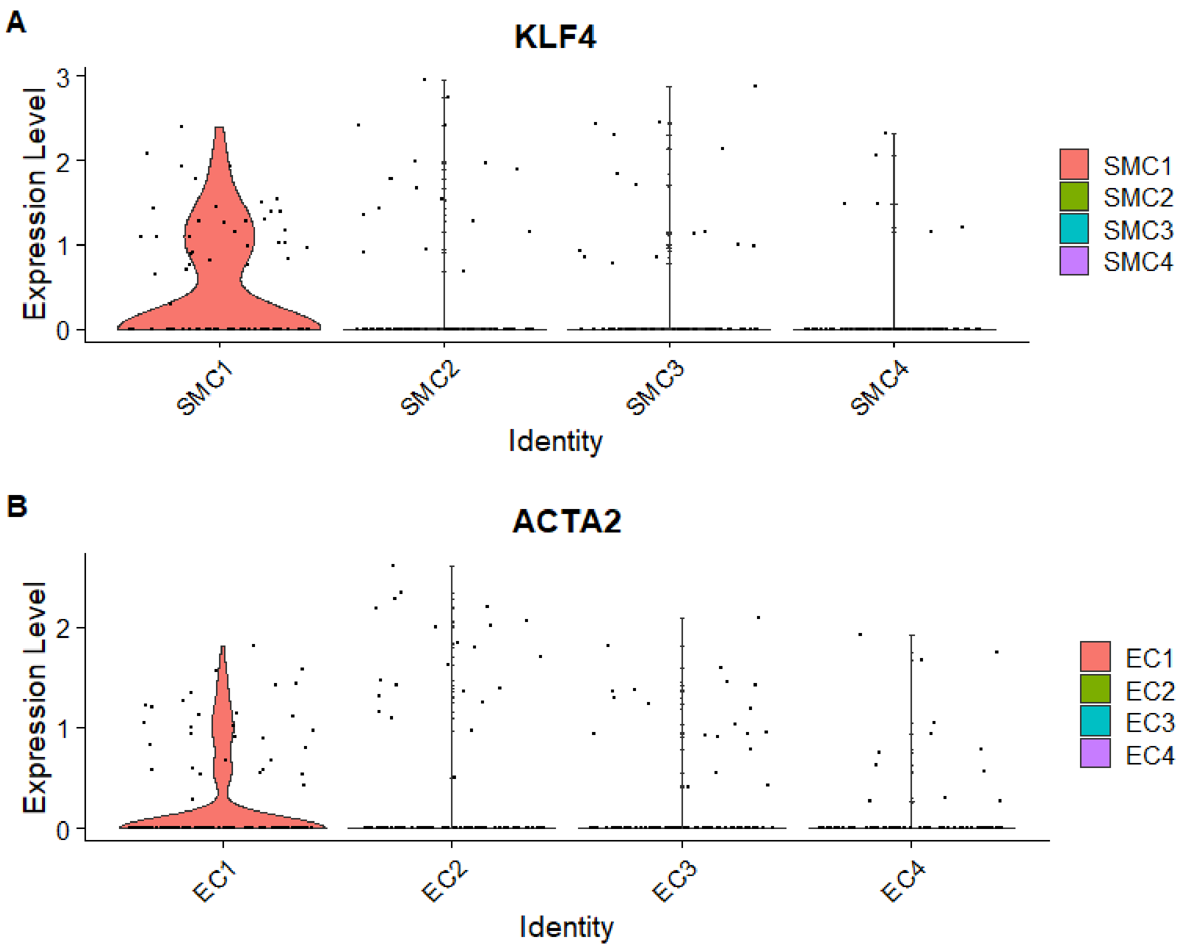

Figure S6. Single-cell expression of genes of interest in smooth muscle and endothelial cells from carotid plaque. A. Expression of *KLF4* gene in SMC subclusters. B. Expression of *ACTA2* in Endothelial Cell subclusters.

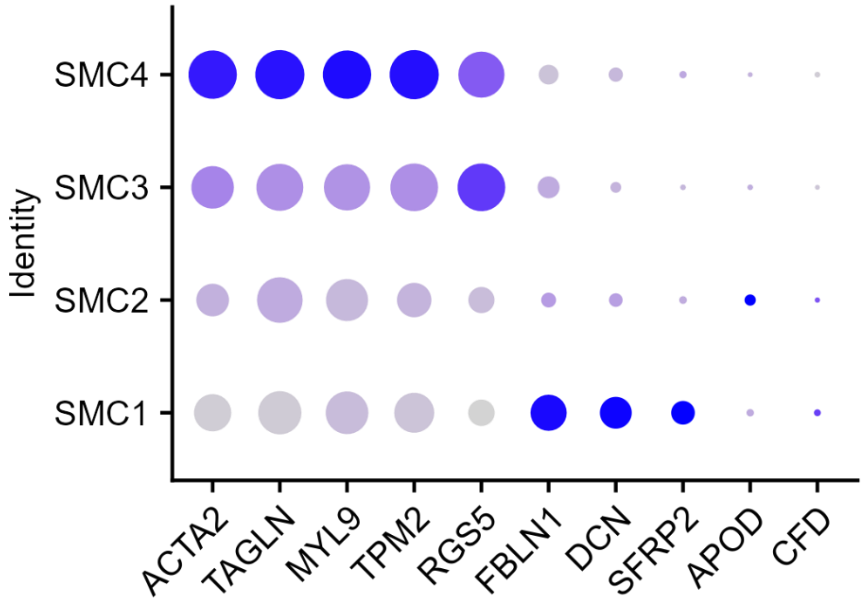

Figure S7. Single-cell expression of Myofibroblast-related genes in smooth muscle cell (SMCs) subtypes from carotid plaque.

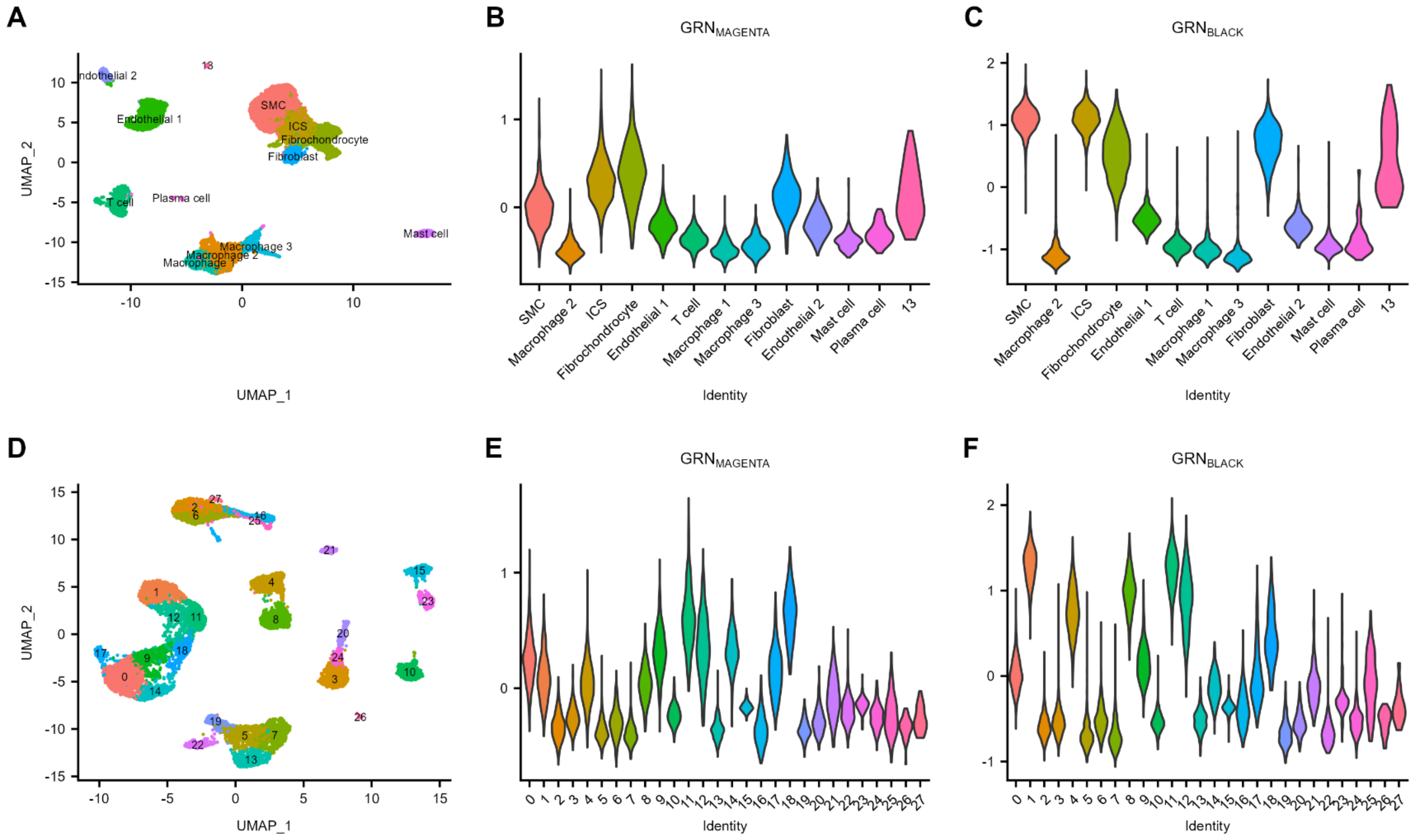

Figure S8. Expression of GRN$_{MAGENTA}$ and GRN$_{BLACK}$ key driver genes in two vascular single cell dataset from carotid plaque[2] and coronaries from heart transplant donors[3]. A. Single-cell clustering of cells from carotid plaque with 13 cell-type clusters. B. Module score for the expression of GRN$_{MAGENTA}$ KD genes in the carotid plaque clusters. C. Module score for the expression of GRN$_{BLACK}$ KD genes in the carotid plaque clusters. D. Single-cell re-clustering of cells from coronary tissue (including plaque) with 28 clusters. E. Module score for the expression of GRN$_{MAGENTA}$ KD genes in the coronary tissue clusters. F. Module score for the expression of GRN$_{BLACK}$ KD genes in the coronary tissue clusters.

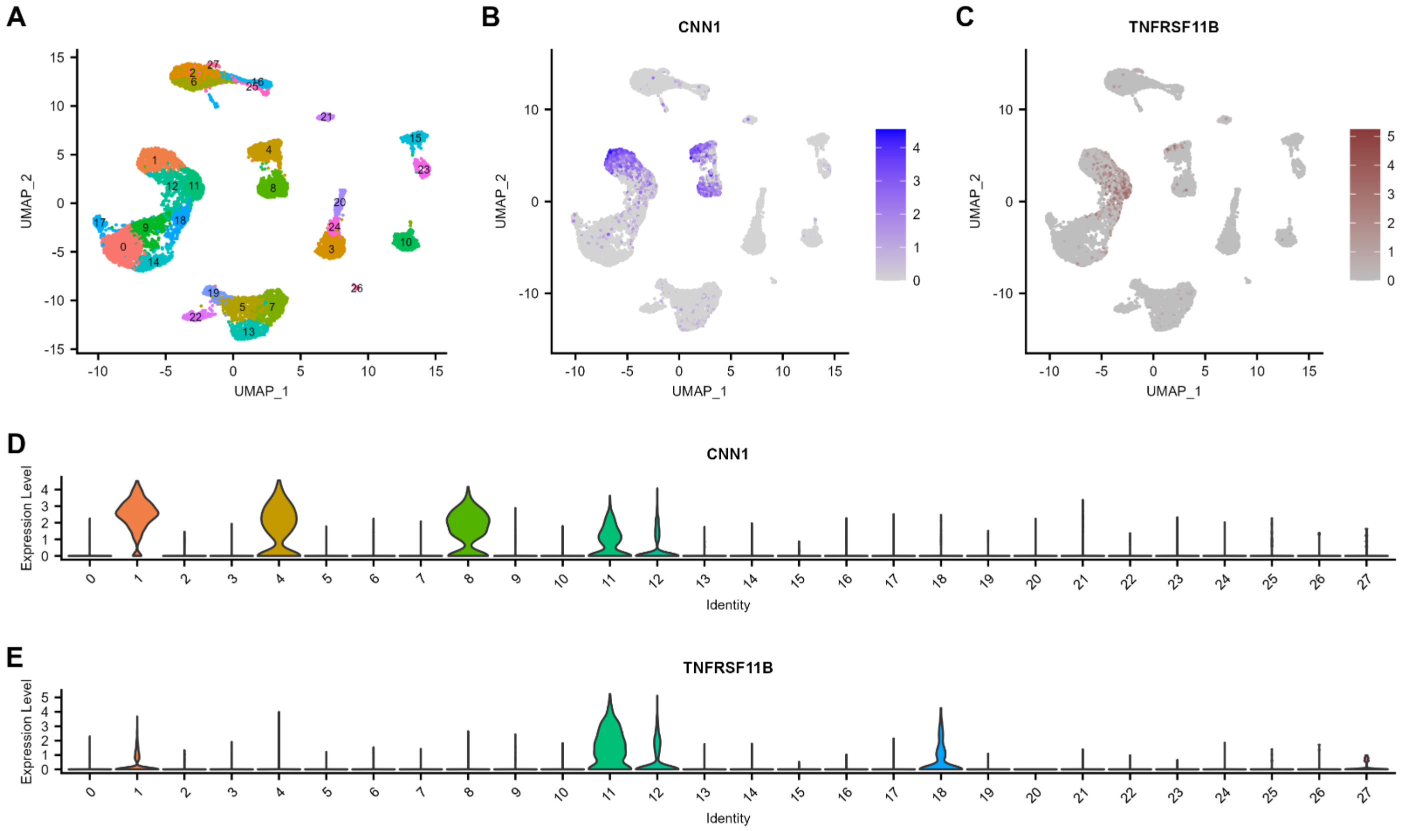

Figure S9. Re-identification of SMC and phenotypically modulated Fibromyocytes in coronary single-cell from heart transplant donors. A. Single-cell re-clustering of cells from coronary tissue (including plaque) with 28 clusters. B. Feature plot representing expression of *CNN1* in the single-cell clusters. C. Feature plot representing expression of *TNFRSF11B* in the single-cell clusters. D. Violin plot representing expression of *CNN1* in the different cell clusters. E. Violin plot representing expression of *TNFRSF11B* in the different cell clusters.

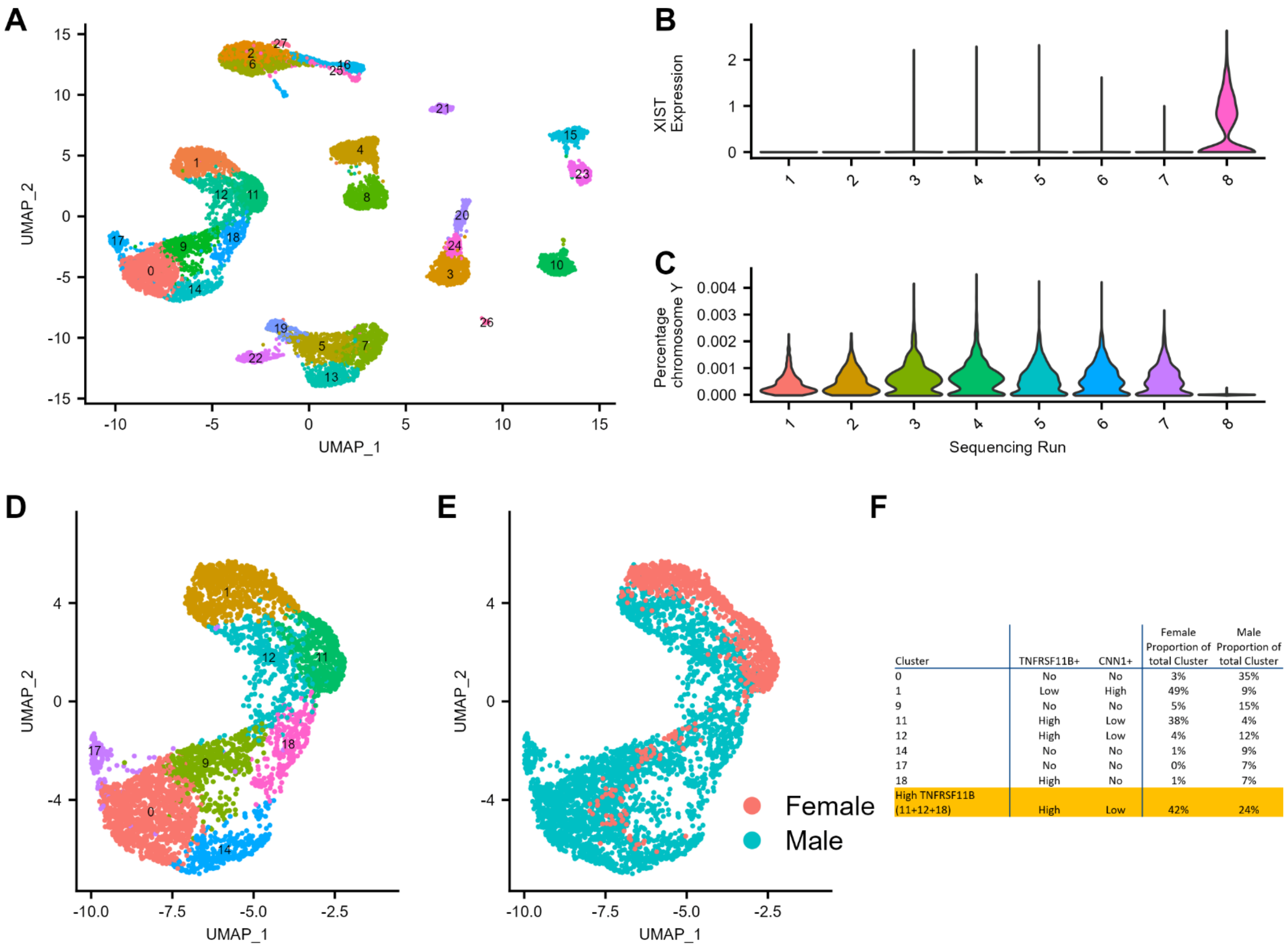

Cluster	TNFRSF11B+	CNN1+	Female Proportion of total Cluster	Male Proportion of total Cluster
0	No	No	3%	35%
1	Low	High	49%	9%
9	No	No	5%	15%
11	High	Low	38%	4%
12	High	Low	4%	12%
14	No	No	1%	9%
17	No	No	0%	7%
18	High	No	1%	7%
High TNFRSF11B (11+12+18)	High	Low	42%	24%

Figure S10. Sex-typing of 11,756 single-cells from coronary tissue from 4 patients (three males, 7 independent sequencing runs in total; and one female)[3] ***supports higher contribution of modulated SMCs to female plaques.*** A. Single-cell UMAP of 11,756 cells from coronary tissue (including plaque) with 28 clusters. B. Violin plot of *XIST* expression for cells within each sequencing run. C. Percentage of transcripts originating from chromosome Y genes in each sequencing run. D. Zoom-in UMAP of SMC-Fibroblast cells (4,212 cells) including 8 clusters from panel. E. Zoom-in UMAP of SMC-Fibroblast cells including 8 clusters from panel A colored by sex using data from panels B and C. F. Table summarizing *TNFRSF11B+* clusters (Figure S9) and their contribution to the SMC-Fibroblast cell population in males and females.

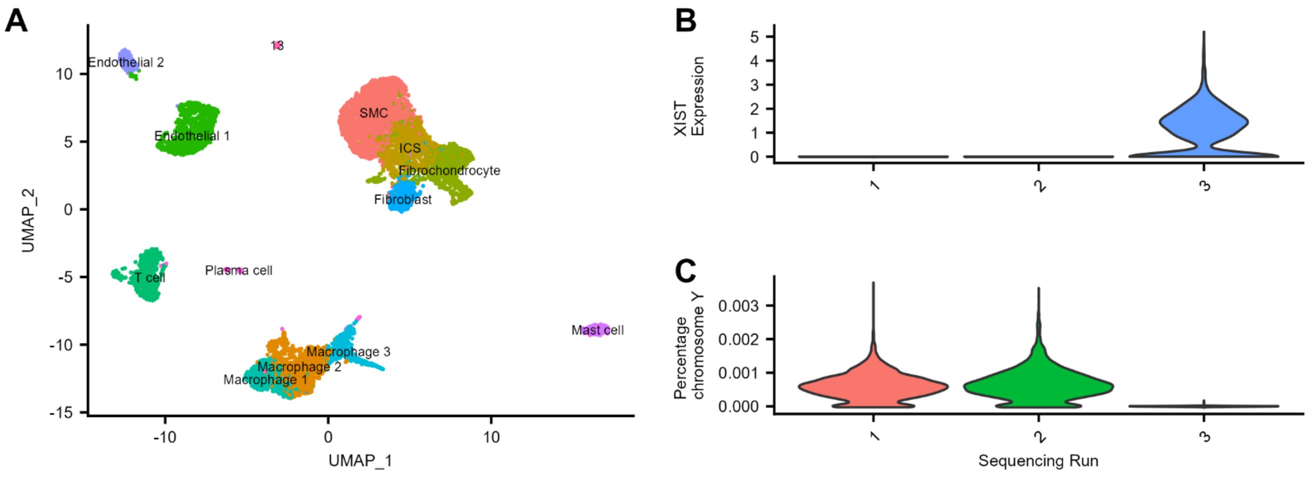

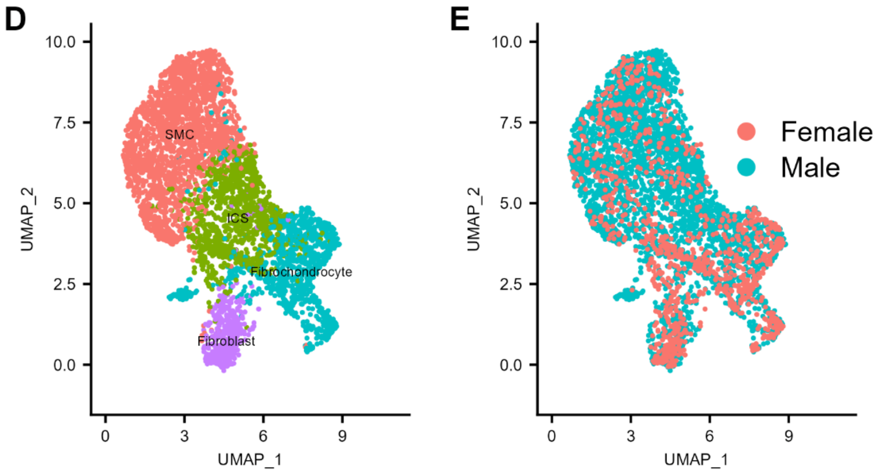

F

Cluster	TNFSFR11B+	CNN1+	Female Proportion of total Cluster	Male Proportion of total Cluster
SMC	Low	High	28%	51%
ICS	High	High	21%	24%
Fibrochondrocyte	High	Low	34%	19%
Fibroblast	Low	Low	17%	6%
High TNFSFR11B+ (ICS + Fibrochondrocyte)	High	Low	55%	43%

Figure S11. Sex-typing of 8,867 single-cells from carotid plaque from 3 patients (two males, and one female)*[2] *supports higher contribution of modulated SMCs to female plaques. A. Single-cell UMAP of 8,867 cells from coronary tissue (including plaque) with 14 clusters. B. Violin plot of *XIST* expression for cells within each sequencing run. C. Percentage of transcripts originating from chromosome Y genes in each sequencing run. D. Zoom-in UMAP of SMC-Fibroblast cells (4,402 cells) including 4 clusters from panel A. E. Zoom-in UMAP of SMC-Fibroblast cells including 4 clusters from panel A colored by sex using data from panels B and C. F. Table summarizing *TNFRSF11B+* modulated SMC (ICS and Fibrochondrocytes) and their contribution to the SMC-Fibroblast cell population in males and females.

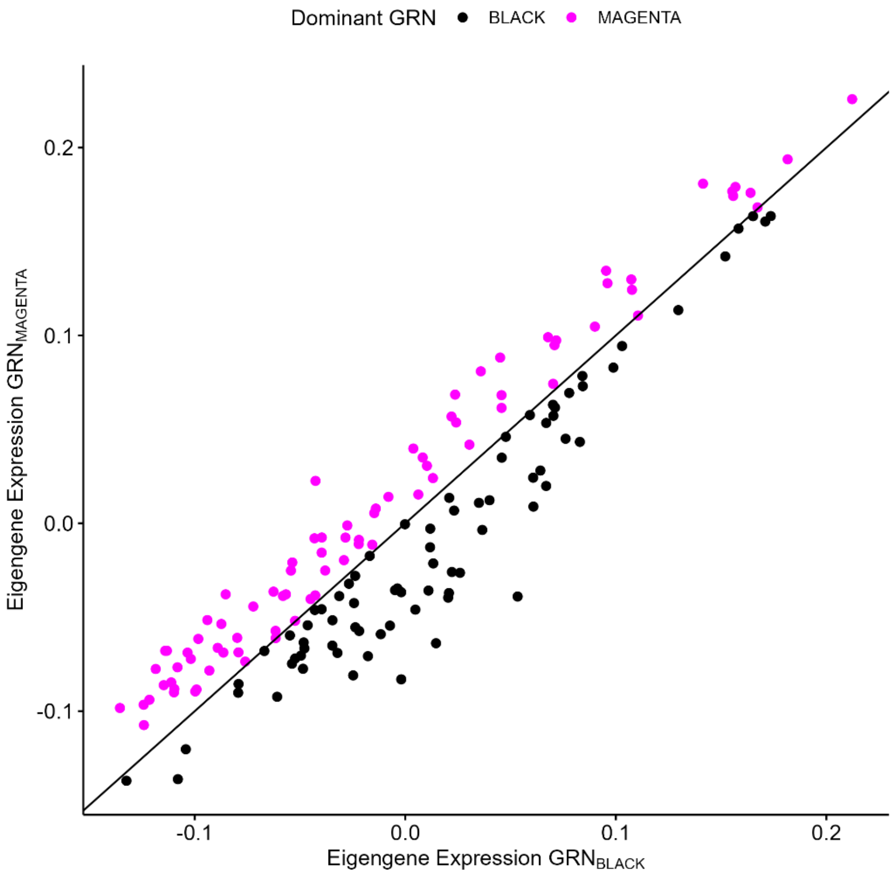

Figure S12. Eigenegene expression of $GRN_{MAGENTA}$ and GRN_{BLACK} in 158 female carotid plaques. Each dot represents a patients plaque and it has been coloured based on the highest expressed network (i.e. black or magenta).

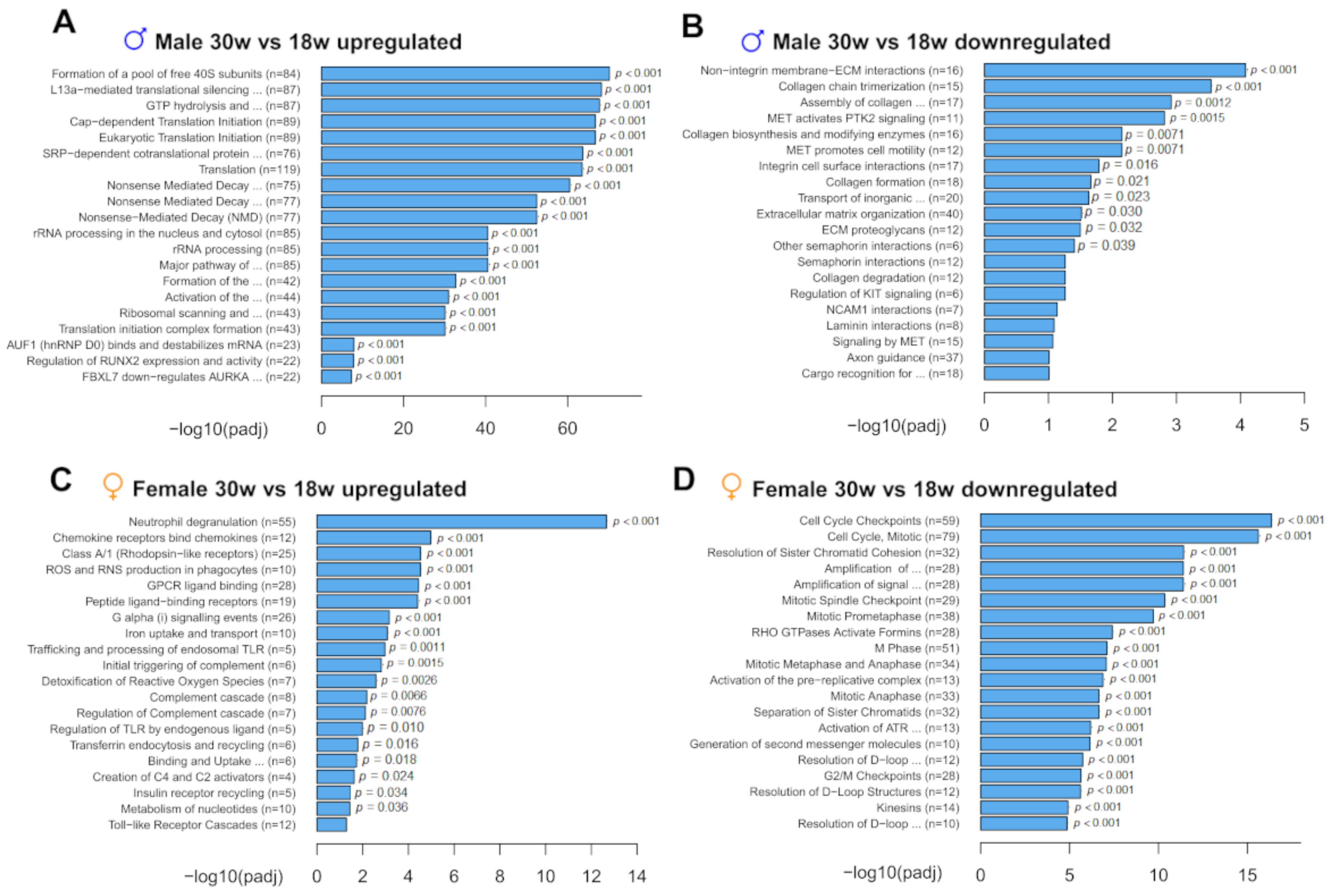

Figure S13. Gene enrichment analysis during atherosclerosis progression in brachiocephalic artery (BCA) lesions from male female and male Apoe-/- mice fed a western diet. A. Reactome pathway enrichment analysis for genes upregulated in 30w vs. 18w of WD in male mice. B. Reactome pathway enrichment analysis for genes

downregulated in 30w vs. 18w of WD in male mice. C. Reactome pathway enrichment analysis for genes upregulated in 30w vs. 18w of WD in female mice. D. Reactome pathway enrichment analysis for genes down-regulated in 30w vs. 18w of WD in female mice.

Major Resources Table

Animals (in vivo studies)

Species	Vendor or Source	Background Strain	Sex	Persistent ID / URL
Mus Musculus (Cdh5-Cre ERT2 R26R-eYFP Apoe-/- mice)	Jackson laboratory	C57BL/6J	M/F	DOI:https://doi.org/10.1038/s42255-020-00338-8

Data & Code Availability

Description	Source / Repository	Persistent ID / URL
AtheroExpress Biobank Database	Dataverse NL	https://doi.org/10.34894/4IKE3T
scRNAseq data Athero Express	Dataverse NL	https://doi.org/10.34894/TYHGEF
Bulk RNAseq data AtheroExpress	Dataverse NL	https://doi.org/10.34894/D1MDKL
